# Anal cancer in high-income countries: Increasing burden of disease

**DOI:** 10.1371/journal.pone.0205105

**Published:** 2018-10-19

**Authors:** Yoon-Jung Kang, Megan Smith, Karen Canfell

**Affiliations:** 1 Cancer Research Division, Cancer Council NSW, Sydney, New south Wales, Australia; 2 School of Public Health, Sydney Medical School, University of Sydney, Sydney, New South Wales, Australia; 3 Prince of Wales Clinical School, University of New South Wales, Sydney, New South Wales, Australia; Leibniz Institute for Prevention Research and Epidemiology BIPS, GERMANY

## Abstract

**Background:**

Previous studies have reported that anal cancer incidence has increased in individual countries; however, age-specific trends were not examined in detail. This study describes pooled and country-specific anal cancer incidence trends by sex, age (all ages, <60 and 60+ years) and histological subtype (all subtypes, squamous cell carcinoma [SCC] and adenocarcinoma [ADC]).

**Methods:**

Five-year incidence and population-at-risk data were obtained from IARC’s *Cancer Incidence in Five Continents* for the years 1988–1992 to 2008–2012. The standardised rate ratios (SRRs) for 2008–2012 vs 1988–1992 and the 5-year average percent change (AvPC) during the period were used to assess changes in the age-standardised incidence rates.

**Results:**

During the study period, there were significant increases in the incidence of SCC in both men and women of all age groups with significant increasing trend, and these increases were highest in those aged <60 years (SRR = 2.34 [95% CI:2.11–2.58] in men and SRR = 2.76 [95% CI:2.54–3.00] in women). By contrast, there were significant decreases in the incidence of ADC in men and women of all ages (SRR = 0.60 [95% CI:0.54–0.67]) and (SRR = 0.63 [95% CI:0.56–0.71], respectively), with similar decreases in those aged <60 years and 60+ years. These competing trends still resulted in significant increases in the overall incidence of anal cancer in men and women of all ages groups with significant increasing trend. The SRRs in men of all ages, <60 years and 60+ years were 1.35 (95% CI:1.28–1.42), 1.77 (95% CI:1.62–1.92) and 1.08 (95% CI:1.00–1.15), respectively. The corresponding SRRs in women were 1.75 (95% CI:1.67–1.83), 2.31 (95% CI:2.14–2.48) and 1.38 (95% CI 1.31–1.46), respectively.

**Conclusion:**

Increases in the incidence of anal SCC has driven an overall increase in anal cancer incidence; this may be associated with changing sexual behaviours and increasing levels of HPV exposure in younger cohorts. The findings further reinforce the importance of HPV vaccination.

## Introduction

Anal cancer is rare in the general population, accounting for 4% of all cancers of the lower gastrointestinal tract. [1] Worldwide, approximately 88% of anal cancer cases are associated with human papillomavirus (HPV) infection [2], with HPV 16 the most commonly detected type, followed by HPV 18. [3, 4] The two main morphologic variants in anal cancer are squamous cell carcinoma (SCC) and adenocarcinoma (ADC). SCC represents ~70% of anal cancer cases and shares many risk factors with cervical cancer, in particular infection with HPV; whereas ADC is less likely to be related to HPV infection. [5, 6] The risk of anal cancer is elevated among men with a history of receptive anal intercourse, women with a history of cervical or vulvar cancer presumably because both are linked to exposure to oncogenic HPV infections, and because of the potential for infections to spread between these sites [6–8], and people with immune deficiency, including those who are infected with Human Immunodeficiency Virus (HIV) and organ transplantation recipients. [9, 10]

Recent studies have reported that anal cancer incidence has increased for both sexes, especially in more developed regions including Canada [11], the USA [12–15], Denmark [16, 17], Sweden [18], Southeast England [19], Scotland [20] and Australia [21]. However, there are inconsistencies in the characteristics of tumours included in these analyses (i.e. included histological subtypes and whether or not *in situ* disease or SCC of the rectum were included) and often age-specific trends were not well documented. Findings from a recent study on trends in anal cancer incidence across all ages in 18 countries up to 2007 [22] were consistent with previous reports in the above-mentioned individual countries that reported the trends across all ages. That study focused on reporting anal cancer incidence trends in as many countries as possible to see whether the increase in the incidence of anal cancer seen in North America, Europe and Oceania were also observed in other geographical areas, but did not report pooled estimates across regions or worldwide, or age-specific trends in incidence.

We have previously reported that the incidence of another HPV-related anogenital cancer, vulvar cancer, has increased in the last 20 years in high-income countries, potentially due to changing sexual behaviours and increasing levels of exposure to HPV in cohorts born around/after 1950. [23, 24] Therefore, the aim of this study was to systematically assess trends in the incidence of anal cancer in all countries for which suitable registry data were available, and to determine if the trends varied by sex, histological subtype, or in different age groups using the latest available data.

## Materials and methods

The current study used similar methods to previous analyses of trends in the incidence of vulvar cancer. [23, 24]

### Data sources

Data in 5-year aggregated blocks by sex and histological subtype were obtained from IARC’s *Cancer Incidence in Five Continents (CI5)* for invasive anal cancer cases diagnosed between 1988–1992 (CI5 Volume 7) and 2008–2012 (CI5 Volume 11). [25, 26] We included all cases classified as C21 according to the 10^th^ revision of the International Classification of Disease (ICD-10). Histological subtypes were grouped into squamous cell carcinoma (SCC; 8050–8076, 8083–8084, 8123–8124), adenocarcinoma (ADC; 8140–8145, 8190–8231, 8260–8263, 8310, 8401, 8480–8490, 8550–8551, 8570–8574, 8576) and all other types including other specified carcinoma, unspecified carcinoma (8010–8011), melanoma (8720–8790), other specified malignant neoplasm and unspecified malignant neoplasm (8000–8005). [27] Unspecified malignant neoplasms comprised <0.3% of the total cases classified as C21. Data on risk factors for anal cancer, such as HPV or HIV status, were not available from this aggregated registry data. Countries were included in the analysis if the available registry data fulfilled several *a priori* conditions regarding reliability, which consequently provided comparability of included registries. These *a priori* conditions were: i) at least one jurisdictional cancer registry in the country covered its entire catchment area and reported for the whole period from 1988 to 2012; ii) information on histological subtype was available; iii) the population at risk in each 5-year age group was available; iv) the reported incidence of SCC and ADC of the anus for the first and the last 5-year period (i.e. 1988–1992 and 2008–2012) was not zero (as this may have potentially indicated under-reporting associated with potential disease misclassification); and v) no obvious signs of over-reporting associated with potential disease misclassification (e.g. age-standardised incidence rate of anal cancer ≥ 5 per 100,000, which may have included ADC arising from the rectum). Since anal cancer is a rare disease we additionally calculated pooled estimates to assess trends by combining data across the countries, whilst quantifying statistical uncertainty by providing 95% confidence intervals for both results presented at a country level and aggregated across countries.

Ethics approval was not sought for this study since publicly available aggregate data were used for the analysis.

### Analysis of trends in the incidence of anal cancer

Trends in anal cancer incidence were examined by sex, histology (all histology, SCC and ADC) and age (all ages, <60 years and 60+ years) in each individual country and geographical area, and across all the included countries. The number of anal cancer cases with unknown age at diagnosis were minimal (0.02% of the total cases) and were excluded from the analysis. Age 60 years at diagnosis was chosen as a surrogate measure of changing sexual behaviours and increasing HPV exposure in people born about 1950 or later. Region-specific and overall incidence rates were calculated by dividing pooled case numbers by pooled population size across a region or across all included countries.

Age-specific incidence rates of anal cancer were calculated in men and women aged 20+ years at diagnosis and the results were stratified by birth cohort to examine trends in the incidence rates in successive birth cohorts. Birth cohort-specific information was analysed for cohorts born every five years between 1900 and 1988. Estimates were based on 5-year groupings of age at diagnosis, and 5-year diagnosis period. Age-standardised rates (ASRs) for cancer incidence were calculated using the Segi 1960 World Standard Population. [28]

To test whether there was a significant change in the ASR for anal cancer incidence over time, we calculated 5-year average percent change (AvPC) using ‘Joinpoint’ regression models (Joinpoint Regression Program, Version 4.3.1.0). [29] We allowed a maximum of one joinpoint over the period and obtained 95% confidence intervals (CI) for the AvPC from the model. We also calculated standardised rate ratios (SRR), which is the ratio of the 5-year average standardised incidence rate at the end of the period (2008–2012) relative to the rate at the beginning of the period (1988–1992), as well as the 95% CIs for the SRRs using Poisson approximation. [30]

## Results

### Review of cancer registry data

Cancer registration data from seven countries satisfied the *a priori* conditions for inclusion in the analysis (Canada, USA, Denmark, France, Netherlands, UK and Australia). Population coverage of the registries included in the analysis (as a percent of the country’s total population) ranged from 6% (USA) to 100% (Denmark, Netherlands), with the majority covering >40% of the population in each country (Supporting information S1 Table).

### Analysis of trends in the incidence of anal cancer

The relative proportion of anal cancer that was SCC (over the whole period and across the seven countries) tended to be lower in men than in women (59% vs. 71%), and in those aged 60+ years than in those <60 years in both men (50% vs. 72%) and women (65% vs. 82%). Correspondingly, the relative proportions of ADC tended to be higher in men than in women (32% vs. 20%), and higher in those aged 60+ years than in those <60 years in both men (41% vs. 20%) and women (25% vs. 12%). These findings were consistent in each individual country and when considered by geographical area, except for Canada where the relative proportion of SCC in males was substantially smaller than in other countries (Table 1). Five-year data on the total number of anal cancer cases and population at risk in each country are provided in the Supporting information S2 Table.

**Table 1:** Number of incident anal cancer cases by age, sex and histological subtype in selected high income countries, 1988–1992 to 2008–2012.

Continent	All ages				<60 years				60+ years			
	All subtypes	SCC	ADC	Others	All subtypes	SCC	ADC	Others	All subtypes	SCC	ADC	Others
***Male***												
***(a) Overall (7 countries including Canada*, *USA*, *4 European countries and Australia)***												
Overall	14515 (100%)	8549 (59%)	4702 (32%)	1264 (9%)	5813 (100%)	4213 (72%)	1166 (20%)	434 (7%)	8702 (100%)	4336 (50%)	3536 (41%)	830 (10%)
***(b) By continent***												
North America	7279 (100%)	4012 (55%)	2670 (37%)	597 (8%)	3090 (100%)	2199 (71%)	660 (21%)	231 (7%)	4189 (100%)	1813 (43%)	2010 (48%)	366 (9%)
Europe	5160 (100%)	3295 (64%)	1377 (27%)	488 (9%)	1939 (100%)	1449 (75%)	339 (17%)	151 (8%)	3221 (100%)	1846 (57%)	1038 (32%)	337 (10%)
Oceania^a^	2076 (100%)	1242 (60%)	655 (32%)	179 (9%)	784 (100%)	565 (72%)	167 (21%)	52 (7%)	1292 (100%)	677 (52%)	488 (38%)	127 (10%)
***(c) By country***												
Canada	3733 (100%)	1642 (44%)	1714 (46%)	377 (10%)	1362 (100%)	824 (60%)	400 (29%)	138 (10%)	2371 (100%)	818 (35%)	1314 (55%)	239 (10%)
USA	3546 (100%)	2370 (67%)	956 (27%)	220 (6%)	1728 (100%)	1375 (80%)	260 (15%)	93 (5%)	1818 (100%)	995 (55%)	696 (38%)	127 (7%)
Denmark	734 (100%)	542 (74%)	131 (18%)	61 (8%)	291 (100%)	241 (83%)	28 (10%)	22 (8%)	443 (100%)	301 (68%)	103 (23%)	39 (9%)
France	504 (100%)	308 (61%)	164 (33%)	32 (6%)	179 (100%)	131 (73%)	36 (20%)	12 (7%)	325 (100%)	177 (54%)	128 (39%)	20 (6%)
The Netherlands	1208 (100%)	895 (74%)	234 (19%)	79 (7%)	490 (100%)	399 (81%)	63 (13%)	28 (6%)	718 (100%)	496 (69%)	171 (24%)	51 (7%)
UK	2714 (100%)	1550 (57%)	848 (31%)	316 (12%)	979 (100%)	678 (69%)	212 (22%)	89 (9%)	1735 (100%)	872 (50%)	636 (37%)	227 (13%)
Australia	2076 (100%)	1242 (60%)	655 (32%)	179 (9%)	784 (100%)	565 (72%)	167 (21%)	52 (7%)	1292 (100%)	677 (52%)	488 (38%)	127 (10%)
***Female***												
***(a) Overall (7 countries including Canada*, *USA*, *4 European countries and Australia)***												
Overall	20779 (100%)	14784 (71%)	4246 (20%)	1749 (8%)	7693 (100%)	6284 (82%)	913 (12%)	496 (6%)	13086 (100%)	8500 (65%)	3333 (25%)	1253 (10%)
***(b) By continent***												
North America	9904 (100%)	6874 (69%)	2230 (23%)	800 (8%)	3801 (100%)	3015 (79%)	529 (14%)	257 (7%)	6103 (100%)	3859 (63%)	1701 (28%)	543 (9%)
Europe	8123 (100%)	5949 (73%)	1467 (18%)	707 (9%)	2899 (100%)	2446 (84%)	280 (10%)	173 (6%)	5224 (100%)	3503 (67%)	1187 (23%)	534 (10%)
Oceania^a^	2752 (100%)	1961 (71%)	549 (20%)	242 (9%)	993 (100%)	823 (83%)	104 (10%)	66 (7%)	1759 (100%)	1138 (65%)	445 (25%)	176 (10%)
***(c) By country***												
Canada	5133 (100%)	3339 (65%)	1283 (25%)	511 (10%)	1910 (100%)	1454 (76%)	286 (15%)	170 (9%)	3223 (100%)	1885 (58%)	997 (31%)	341 (11%)
USA	4771 (100%)	3535 (74%)	947 (20%)	289 (6%)	1891 (100%)	1561 (83%)	243 (13%)	87 (5%)	2880 (100%)	1974 (69%)	704 (24%)	202 (7%)
Denmark	1493 (100%)	1223 (82%)	147 (10%)	123 (8%)	595 (100%)	541 (91%)	24 (4%)	30 (5%)	898 (100%)	682 (76%)	123 (14%)	93 (10%)
France	1235 (100%)	1015 (82%)	150 (12%)	70 (6%)	372 (100%)	329 (88%)	32 (9%)	11 (3%)	863 (100%)	686 (79%)	118 (14%)	59 (7%)
The Netherlands	1577 (100%)	1198 (76%)	283 (18%)	96 (6%)	623 (100%)	554 (89%)	44 (7%)	25 (4%)	954 (100%)	644 (68%)	239 (25%)	71 (7%)
UK	3818 (100%)	2513 (66%)	887 (23%)	418 (11%)	1309 (100%)	1022 (78%)	180 (14%)	107 (8%)	2509 (100%)	1491 (59%)	707 (28%)	311 (12%)
Australia	2752 (100%)	1961 (71%)	549 (20%)	242 (9%)	993 (100%)	823 (83%)	104 (10%)	66 (7%)	1759 (100%)	1138 (65%)	445 (25%)	176 (10%)

SCC–squamous cell carcinoma; ADC–adenocarcinoma.

^a^ Oceania includes Australia only.

**Fig 1:**
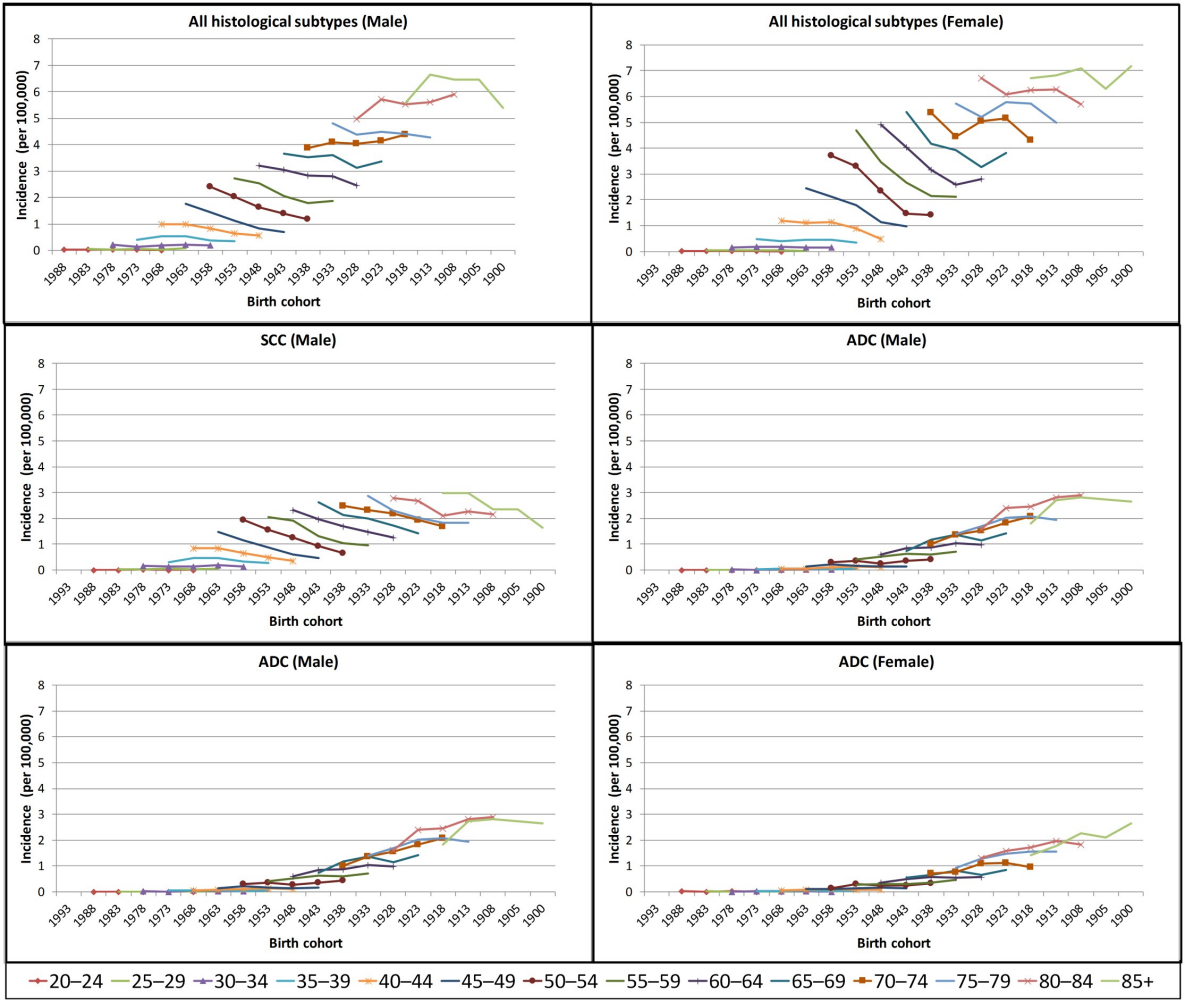
Pooled age-specific anal cancer incidence rates by birth cohort in men and women born from 1900 to 1983, for each of the five 5-yearly average rates (1988–92, 1993–97, 1998–2002, 2003–2007, 2008–2012). SCC–squamous cell carcinoma; ADC–adenocarcinoma; M–male; F- female.

**Fig 2:**
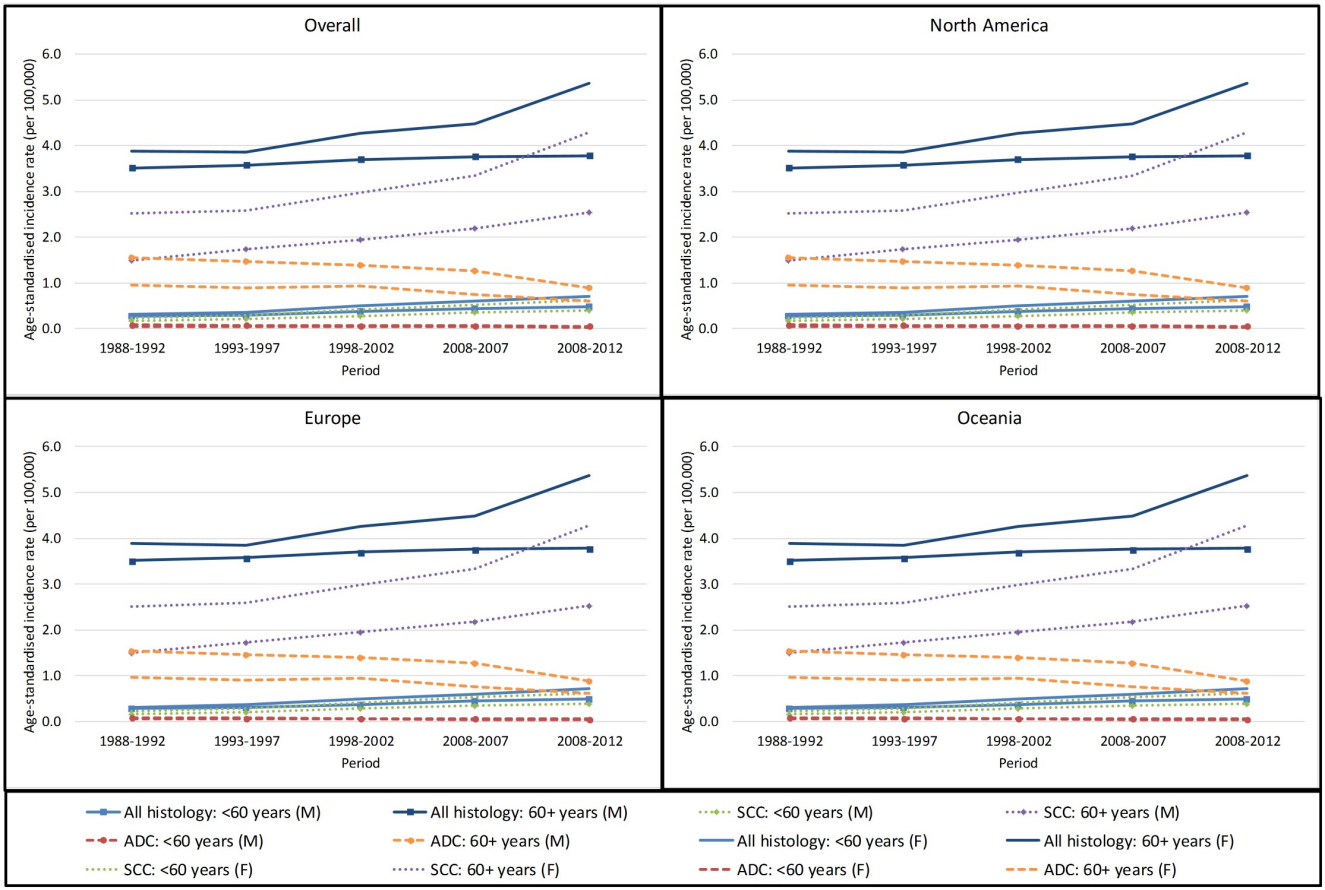
Pooled anal cancer incidence rates in selected high income countries, by region, sex, histological subtype and age group. Note) Oceania includes Australia only. SCC–squamous cell carcinoma; ADC–adenocarcinoma; M–male; F- female.

As expected, in both men and women, the incidence of anal cancer increased with increasing age for all birth cohorts examined, when combining all histological subtypes and separately for SCC and ADC (Fig 1). Age-specific incidence rates by histological subtype and geographical area are illustrated in S1 and S2 Figs. The age-standardised incidence rates are shown in Fig 2.

The incidence of anal cancer across all ages and histological subtypes was significantly higher in 2008–2012 than in 1988–1992, and increased across the period under consideration (SRR = 1.35; 5-year AvPC = 8.3% [p = 0.001] in men; SRR = 1.75; 5-year AvPC = 15.8% [p = 0.001] in women) (Table 2). Rates increased in both those aged <60 years and 60+ years, with significant increasing trend. The increases were highest in those aged <60 years in both male and female. The SRR and the 5-year AvPC were significantly elevated in each geographical region in men and women of all ages and aged <60 years. Considering each country separately, significant increases in the SRRs in 2008–2012 relative to 1988–1992 as well as the 5-year AvPC between 1988–1992 and 2008–2012 were seen in the USA, the Netherlands, the UK and Australia, in both men and women aged <60 years. In Canada and France, the incidence in male 60+ years in 2008–2012 compared to 1988–1992 decreased but the trend was not significant.

**Table 2:** Age-standardised anal cancer incidence rates (per 100,000 individuals), 5-year average percent change and standardised rate ratios compared to 1988–1992 in selected high income countries: All histological types.

Sex	Continent/Country	All ages				<60 years				60+ years			
		ASR^a^ (1988–1992)	ASR^a^ (2008–2012)	5-year AvPC^b^ (95% CI) P value	SRR (95% CI) 2008–2012 vs 1988–1992	ASR^a^ (1988–1992)	ASR^a^ (2008–2012)	5-year AvPC^b^ (95% CI) P value	SRR (95% CI) 2008–2012 vs 1988–1992	ASR^a^ (1988–1992)	ASR^a^ (2008–2012)	5-year AvPC^b^ (95% CI) P value	SRR (95% CI) 2008–2012 vs 1988–1992
***Male***	***(a) Overall (7 countries including Canada*, *USA*, *4 European countries and Australia)***												
	Overall	0.63	0.85	8.3% (6.2%, 10.4%) *P = 0*.*001*	1.35 (1.28–1.42)	0.28	0.49	16.4% (12.2%, 20.8%) *P = 0*.*001*	1.77 (1.62–1.92)	3.51	3.78	2.0% (1.0%, 2.9%) *P = 0*.*01*	1.08 (1.00–1.15)
	***(b) By continent***												
	North America	0.76	0.97	6.9% (1.0%, 13.2%) *P = 0*.*03*	1.28 (1.18–1.38)	0.33	0.59	15.3% (8.9%, 22.1%) *P = 0*.*004*	1.77 (1.57–1.98)	4.23	4.08	0.0% (-6.4%, 6.8%) *P = 1*.*0*	0.97 (0.87–1.07)
	Europe	0.51	0.71	9.0% (3.4%, 15.0%) *P = 0*.*01*	1.39 (1.27–1.51)	0.21	0.38	17.7% (11.4%, 24.2%) *P = 0*.*002*	1.78 (1.54–2.07)	2.93	3.38	3.1% (-4.2%, 10.9%) *P = 0*.*3*	1.15 (1.03–1.29)
	Oceania^c^	0.64	0.90	9.3% (6.6%, 12.1%) *P = 0*.*001*	1.41 (1.22–1.63)	0.31	0.50	15.1% (6.0%, 24.9%) *P = 0*.*01*	1.62 (1.30–2.02)	3.31	4.16	5.0% (-3.9%, 14.7%) *P = 0*.*2*	1.26 (1.04–1.51)
	***(c) By country***												
	Canada	0.85	0.85	1.1% (-8.4%, 11.5%) *P = 0*.*8*	0.99 (0.89–1.11)	0.29	0.43	9.9% (-2.4%, 23.7%) *P = 0*.*1*	1.47 (1.23–1.77)	5.34	4.18	-4.4% (-13.3%, 5.4%) *P = 0*.*2*	0.78 (0.68–0.90)
	USA	0.67	1.09	13.0% (10.4%, 15.6%) *P = 0*.*0004*	1.62 (1.45–1.80)	0.37	0.73	19.4% (15.8%, 23.2%) *P = 0*.*0004*	1.99 (1.72–2.31)	3.15	3.99	5.9% (2.5%, 9.3%) *P = 0*.*01*	1.27 (1.08–1.48)
	Denmark	0.53	0.82	10.3% (5.4%, 15.5%) *P = 0*.*01*	1.54 (1.22–1.96)	0.20	0.45	16.2% (-1.4%, 37.0%) *P = 0*.*1*	2.29 (1.51–3.47)	3.26	3.84	4.5% (-6.8%, 17.2%) *P = 0*.*3*	1.18 (0.89–1.56)
	France	0.69	0.59	-4.1% (-13.8%, 6.7%) *P = 0*.*3*	0.86 (0.64–1.16)	0.25	0.33	9.1% (0.7%, 18.1%) *P = 0*.*04*	1.31 (0.80–2.14)	4.18	2.68	-12.9% (-25.8%, 2.3%) *P = 0*.*1*	0.64 (0.44–0.93)
	The Netherlands	0.34	0.60	19.7% (5.8%, 35.3%) *P = 0*.*02*	1.77 (1.48–2.13)	0.17	0.31	21.9% (2.3%, 45.3%) *P = 0*.*04*	1.80 (1.36–2.39)	1.68	2.95	17.8% (6.7%, 29.9%) *P = 0*.*01*	1.75 (1.38–2.22)
	UK	0.57	0.78	7.8% (3.3%, 12.4%) *P = 0*.*01*	1.37 (1.21–1.55)	0.24	0.43	17.8% (11.1%, 25.0%) *P = 0*.*003*	1.81 (1.47–2.23)	3.27	3.64	1.0% (-5.3%, 7.7%) *P = 0*.*7*	1.12 (0.96–1.30)
	Australia	0.64	0.90	9.3% (6.6%, 12.1%) *P = 0*.*001*	1.41 (1.22–1.63)	0.31	0.50	15.1% (6.0%, 24.9%) *P = 0*.*01*	1.62 (1.30–2.02)	3.31	4.16	5.0% (-3.9%, 14.7%) *P = 0*.*2*	1.26 (1.04–1.51)
***Female***	***(a) Overall (7 countries including Canada*, *USA*, *4 European countries and Australia)***												
	Overall	0.70	1.22	15.8% (12.0%, 19.7%) *P = 0*.*001*	1.75 (1.67–1.83)	0.31	0.71	23.8% (18.0%, 30.0%) *P = 0*.*001*	2.31 (2.14–2.48)	3.88	5.37	8.9% (3.0%, 15.2%) *P = 0*.*02*	1.38 (1.31–1.46)
	***(b) By continent***												
	North America	0.86	1.32	13.0% (8.1%, 18.1%) *P = 0*.*003*	1.54 (1.44–1.65)	0.38	0.74	19.8% (11.1%, 29.2%) *P = 0*.*005*	1.98 (1.78–2.19)	4.75	6.00	7.2% (1.6%, 13.1%) *P = 0*.*03*	1.26 (1.16–1.37)
	Europe	0.56	1.13	18.7% (13.4%, 24.2%) *P = 0*.*001*	2.02 (1.88–2.18)	0.24	0.67	27.9% (20.3%, 35.9%) *P = 0*.*001*	2.84 (2.51–3.21)	3.15	4.82	10.6% (2.9%, 18.9%) *P = 0*.*02*	1.53 (1.40–1.68)
	Oceania^c^	0.72	1.22	16.1% (5.6%, 27.6%) *P = 0*.*02*	1.70 (1.50–1.92)	0.33	0.72	25.3% (8.8%, 44.2%) *P = 0*.*01*	2.18 (1.79–2.66)	3.85	5.25	8.7% (2.4%, 15.3%) *P = 0*.*02*	1.36 (1.17–1.59)
	***(c) By country***												
	Canada	0.85	1.28	12.0% (7.3%, 16.9%) *P = 0*.*004*	1.51 (1.37–1.66)	0.38	0.71	19.2% (8.2%, 31.2%) *P = 0*.*01*	1.87 (1.61–2.17)	4.64	5.89	6.4% (3.2%, 9.6%) *P = 0*.*01*	1.27 (1.13–1.43)
	USA	0.87	1.36	13.8% (7.8%, 20.2%) *P = 0*.*005*	1.57 (1.43–1.72)	0.37	0.77	20.4% (12.0%, 29.4%) *P = 0*.*004*	2.08 (1.80–2.40)	4.87	6.10	7.7% (-2.8%, 19.4%) *P = 0*.*1*	1.25 (1.12–1.40)
	Denmark	0.73	1.73	20.9% (11.1%, 31.5%) *P = 0*.*01*	2.37 (1.97–2.84)	0.37	1.07	27.2% (8.8%, 48.7%) *P = 0*.*02*	2.91 (2.19–3.87)	3.68	7.10	14.0% (1.7%, 27.9%) *P = 0*.*0*	1.93 (1.53–2.43)
	France	1.05	1.40	6.9% (0.9%, 13.3%) *P = 0*.*04*	1.33 (1.08–1.63)	0.45	0.80	16.5% (11.4%, 21.9%) *P = 0*.*0002*	1.76 (1.25–2.48)	5.90	6.22	-0.2% (-8.7%, 9.1%) *P = 0*.*9*	1.05 (0.82–1.35)
	The Netherlands	0.30	0.68	22.9% (20.3%, 25.5%) *P = 0*.*0001*	2.28 (1.91–2.72)	0.10	0.44	37.8% (24.6%, 52.3%) *P = 0*.*002*	4.41 (3.28–5.92)	1.89	2.60	9.3% (-0.1%, 19.5%) *P = 0*.*05*	1.37 (1.10–1.72)
	UK	0.56	1.27	21.6% (13.9%, 29.8%) *P = 0*.*002*	2.27 (2.04–2.52)	0.24	0.74	29.1% (19.2%, 39.9%) *P = 0*.*0002*	3.06 (2.56–3.66)	3.13	5.55	14.9% (6.4%, 24.1%) *P = 0*.*01*	1.78 (1.56–2.02)
	Australia	0.72	1.22	16.1% (5.6%, 27.6%) *P = 0*.*02*	1.70 (1.50–1.92)	0.33	0.72	25.3% (8.8%, 44.2%) *P = 0*.*01*	2.18 (1.79–2.66)	3.85	5.25	8.7% (2.4%, 15.3%) *P = 0*.*02*	1.36 (1.17–1.59)

ASR–age standardised rate; SRR–standardised rate ratio (compared to 1988–1992); 5-year AvPC–average percent change between successive 5-year periods.

^a^ Incidence rates were age-standardised using the Segi 1960 World Standard Population.

^b^ 5-year average percent change in the standardised incidence rates was estimated over the period 1988–1992, 1993–1997, 1998–2002, 2003–2007 and 2008–2012. Negative signs indicate decrease in the age-standardised rates over time.

^c^ Oceania includes Australia only.

Note) Data are only included in the current analysis from cancer registries that reported for the entire period 1988–2012 and that also satisfied a priori conditions (see Materials and methods). Therefore, the age-standardised incidence rate of each country reported in the above table does not necessarily correspond to each country’s national statistics.

The incidence of SCC of the anus was significantly higher in 2008–2012 compared to 1988–1992 in men and women both aged <60 years and 60+ years, with significant increasing trend across the countries as well as in each geographical area (Table 3). In both men and women these increases were highest in those aged <60 years (SRR = 2.34 and SRR = 2.76, respectively). Relatively larger increases in the SCC incidence were seen in males 60+ years and females <60 years in The Netherlands. However, excluding The Netherlands from the pooled analysis did not change the results substantially; SRR in 2008–2012 compared to 1988–1992 changed from 1.69 (95% CI: 1.54–1.86) to 1.59 (95% CI: 1.44–1.76) in male 60+ years and from 2.76 (95% CI: 2.54–3.00) to 2.63 (95% CI: 2.41–2.86) in females <60 years across the seven countries. In a sensitivity analysis exploring a different age cut-off of 50 years, we found the SRRs for anal SCC incidence in both men and women were similar, but the magnitude of the increase became smaller when using 70 years as an age cut-off (detailed results not shown).

**Table 3:** Age-standardised incidence rates (per 100,000 individuals), 5-year average percent change and standardised rate ratios compared to 1988–1992 in selected high income countries: Squamous cell carcinoma of the anus.

Sex	Continent/Country	All ages				<60 years				60+ years			
		ASR^a^ (1988–1992)	ASR^a^ (2008–2012)	5-year AvPC^b^ (95% CI) P value	SRR (95% CI) 2008–2012 vs 1988–1992	ASR^a^ (1988–1992)	ASR^a^ (2008–2012)	5-year AvPC^b^ (95% CI) P value	SRR (95% CI) 2008–2012 vs 1988–1992	ASR^a^ (1988–1992)	ASR^a^ (2008–2012)	5-year AvPC^b^ (95% CI) P value	SRR (95% CI) 2008–2012 vs 1988–1992
***Male***	***(a) Overall (7 countries including Canada*, *USA*, *4 European countries and Australia)***												
	Overall	0.31	0.63	18.9% (15.6%, 22.2%) *P = 0*.*0003*	2.00 (1.87–2.14)	0.17	0.39	23.6% (15.9%, 31.9%) *P = 0*.*002*	2.34 (2.11–2.58)	1.50	2.53	13.8% (12.4%, 15.2%) *P = 0*.*0001*	1.69 (1.54–1.86)
	***(b) By continent***												
	North America	0.37	0.68	15.4% (9.5%, 21.7%) *P = 0*.*003*	1.86 (1.68–2.05)	0.20	0.46	20.5% (10.2%, 31.8%) *P = 0*.*007*	2.28 (1.99–2.61)	1.70	2.48	8.9% (5.6%, 12.4%) *P = 0*.*003*	1.45 (1.26–1.68)
	Europe	0.24	0.57	23.7% (21.8%, 25.6%) *P = 0*.*00003*	2.32 (2.07–2.59)	0.12	0.32	29.0% (18.8%, 40.0%) *P = 0*.*002*	2.67 (2.23–3.19)	1.27	2.59	19.1% (13.7%, 24.7%) *P = 0*.*001*	2.05 (1.77–2.37)
	Oceania^c^	0.36	0.61	17.1% (6.9%, 28.4%) *P = 0*.*01*	1.69 (1.41–2.03)	0.20	0.38	21.0% (6.8%, 37.1%) *P = 0*.*02*	1.85 (1.43–2.40)	1.63	2.49	13.2% (1.8%, 25.8%) *P = 0*.*03*	1.53 (1.19–1.97)
	***(c) By country***												
	Canada	0.28	0.47	11.0% (1.9%, 20.9%) *P = 0*.*03*	1.65 (1.40–1.95)	0.14	0.28	14.1% (-0.6%, 31.0%) *P = 0*.*1*	1.96 (1.54–2.49)	1.43	2.01	7.4% (0.6%, 14.7%) *P = 0*.*04*	1.41 (1.12–1.76)
	USA	0.45	0.89	18.0% (13.0%, 23.4%) *P = 0*.*001*	1.98 (1.75–2.24)	0.26	0.64	23.9% (15.6%, 32.8%) *P = 0*.*002*	2.44 (2.06–2.87)	1.98	2.95	10.0% (7.6%, 12.5%) *P = 0*.*001*	1.49 (1.23–1.81)
	Denmark	0.34	0.69	19.7% (13.4%, 26.3%) *P = 0*.*002*	2.03 (1.54–2.69)	0.15	0.36	20.2% (-4.0%, 50.4%) *P = 0*.*1*	2.47 (1.55–3.96)	1.88	3.30	18.0% (2.2%, 36.2%) *P = 0*.*03*	1.75 (1.25–2.47)
	France	0.24	0.48	16.4% (5.7%, 28.1%) *P = 0*.*02*	1.97 (1.31–2.96)	0.11	0.28	26.5% (6.5%, 50.1%) *P = 0*.*02*	2.53 (1.34–4.80)	1.31	2.07	7.4% (-6.2%, 23.0%) *P = 0*.*2*	1.58 (0.93–2.67)
	The Netherlands	0.18	0.52	37.5% (17.2%, 61.2%) *P = 0*.*01*	2.87 (2.31–3.57)	0.11	0.28	36.0% (10.4%, 67.5%) *P = 0*.*02*	2.62 (1.90–3.61)	0.78	2.45	38.7% (23.8%, 55.5%) *P = 0*.*003*	3.16 (2.35–4.25)
	UK	0.25	0.58	20.4% (13.4%, 27.7%) *P = 0*.*002*	2.30 (1.95–2.71)	0.12	0.34	28.5% (20.2%, 37.4%) *P = 0*.*001*	2.85 (2.19–3.70)	1.35	2.57	28.5% (20.2%, 37.4%) *P = 0*.*02*	1.91 (1.55–2.34)
	Australia	0.36	0.61	17.1% (6.9%, 28.4%) *P = 0*.*01*	1.69 (1.41–2.03)	0.20	0.38	21.0% (6.8%, 37.1%) *P = 0*.*02*	1.85 (1.43–2.40)	1.63	2.49	13.2% (1.8%, 25.8%) *P = 0*.*03*	1.53 (1.19–1.97)
***Female***	***(a) Overall (7 countries including Canada*, *USA*, *4 European countries and Australia)***												
	Overall	0.48	1.03	21.9% (18.8%, 24.9%) *P = 0*.*0001*	2.15 (2.04–2.26)	0.23	0.62	28.4% (20.5%, 36.8%) *P = 0*.*001*	2.76 (2.54–3.00)	2.51	4.28	15.2% (7.7%, 23.2%) *P = 0*.*007*	1.70 (1.59–1.82)
	***(b) By continent***												
	North America	0.58	1.08	17.5% (13.6%, 21.6%) *P = 0*.*001*	1.84 (1.71–1.98)	0.27	0.63	23.0% (11.1%, 36.2%) *P = 0*.*008*	2.36 (2.10–2.65)	3.16	4.70	11.9% (4.3%, 20.0%) *P = 0*.*01*	1.49 (1.35–1.64)
	Europe	0.37	0.99	26.6% (20.7%, 32.9%) *P = 0*.*001*	2.67 (2.45–2.91)	0.18	0.62	33.8% (23.4%, 45.1%) *P = 0*.*001*	3.52 (3.07–4.03)	1.94	3.99	19.1% (10.7%, 28.1%) *P = 0*.*005*	2.06 (1.84–2.29)
	Oceania^c^	0.52	1.00	22.0% (5.8%, 40.7%) *P = 0*.*02*	1.92 (1.67–2.22)	0.27	0.64	30.6% (10.0%, 55.1%) *P = 0*.*02*	2.40 (1.94–2.97)	2.58	3.94	13.6% (0.3%, 28.8%) *P = 0*.*05*	1.53 (1.26–1.84)
	***(c) By country***												
	Canada	0.48	0.92	17.3% (11.1%, 23.8%) *P = 0*.*003*	1.91 (1.70–2.14)	0.23	0.54	22.3% (6.8%, 40.1%) *P = 0*.*02*	2.35 (1.96–2.81)	2.53	3.99	12.2% (7.2%, 17.3%) *P = 0*.*004*	1.58 (1.36–1.84)
	USA	0.68	1.22	17.8% (11.9%, 24.0%) *P = 0*.*002*	1.80 (1.63–1.98)	0.30	0.71	23.6% (13.1%, 35.0%) *P = 0*.*005*	2.35 (2.02–2.75)	3.75	5.39	11.7% (-0.7%, 25.7%) *P = 0*.*06*	1.44 (1.26–1.63)
	Denmark	0.58	1.54	24.5% (15.2%, 34.6%) *P = 0*.*003*	2.64 (2.16–3.23)	0.32	0.99	30.0% (7.9%, 56.6%) *P = 0*.*02*	3.12 (2.30–4.22)	2.73	5.99	17.6% (2.6%, 34.9%) *P = 0*.*03*	2.19 (1.69–2.85)
	France	0.72	1.26	14.2% (7.5%, 21.2%) *P = 0*.*01*	1.75 (1.39–2.21)	0.33	0.73	22.9% (15.9%, 30.4%) *P = 0*.*002*	2.19 (1.51–3.20)	3.85	5.54	7.0% (-4.3%, 19.5%) *P = 0*.*1*	1.44 (1.08–1.91)
	The Netherlands	0.17	0.60	35.3% (31.1%, 39.7%) *P = 0*.*0001*	3.54 (2.87–4.36)	0.06	0.41	46.2% (25.8%, 69.9%) *P = 0*.*004*	6.50 (4.65–9.09)	1.04	2.15	21.7% (12.7%, 31.5%) *P = 0*.*004*	2.07 (1.58–2.71)
	UK	0.35	1.08	30.6% (20.9%, 41.1%) *P = 0*.*002*	3.07 (2.70–3.48)	0.17	0.66	35.5% (23.9%, 48.3%) *P = 0*.*002*	3.87 (3.16–4.72)	1.83	4.51	25.2% (13.5%, 38.1%) *P = 0*.*01*	2.47 (2.11–2.88)
	Australia	0.52	1.00	22.0% (5.8%, 40.7%) *P = 0*.*02*	1.92 (1.67–2.22)	0.27	0.64	30.6% (10.0%, 55.1%) *P = 0*.*02*	2.40 (1.94–2.97)	2.58	3.94	13.6% (0.3%, 28.8%) *P = 0*.*05*	1.53 (1.26–1.84)

ASR–age standardised rate; SRR–standardised rate ratio (compared to 1988–1992); 5-year AvPC–average percent change between successive 5-year periods.

^a^ Incidence rates were age-standardised using the Segi 1960 World Standard Population.

^b^ 5-year average percent change in the standardised incidence rates was estimated over the period 1988–1992, 1993–1997, 1998–2002, 2003–2007 and 2008–2012. Negative signs indicate decrease in the age-standardised rates over time.

^c^ Oceania includes Australia only.

Note) Data are only included in the current analysis from cancer registries that reported for the entire period 1988–2012 and that also satisfied a priori conditions (see Materials and methods). Therefore, the age-standardised incidence rate of each country reported in the above table does not necessarily correspond to each country’s national statistics.

By contrast, significant decreases in the age-standardised incidence rates of ADC of the anus in 2008–2012 compared to 1988–1992 were observed in men and women both aged <60 years and 60+ years (~40%) across all the seven countries (Table 4) and a significant trend was seen in men and women aged <60 years. In North America and Europe, the incidence of ADC in male and female aged 60+ years significantly decreased.

**Table 4:** Age-standardised incidence rates (per 100,000 individuals), 5-year average percent change and the standardised rate ratios compared to 1988–1992 in selected high income countries: Adenocarcinoma of the anus.

Sex	Continent/ Country	All ages				<60 years				60+ years			
		ASR^a^ (1988–1992)	ASR^a^ (2008–2012)	5-year AvPC^b^ (95% CI) P value	SRR (95% CI) 2008–2012 vs 1988–1992	ASR^a^ (1988–1992)	ASR^a^ (2008–2012)	5-year AvPC^b^ (95% CI) P value	SRR (95% CI) 2008–2012 vs 1988–1992	ASR^a^ (1988–1992)	ASR^a^ (2008–2012)	5-year AvPC^b^ (95% CI) P value	SRR (95% CI) 2008–2012 vs 1988–1992
***Male***	***(a) Overall (7 countries including Canada*, *USA*, *4 European countries and Australia)***												
	Overall	0.24	0.14	-10.6% (-18.7%, -1.7%) *P = 0*.*03*	0.60 (0.54–0.67)	0.08	0.05	-8.2% (-14.5%, -1.5%) *P = 0*.*03*	0.66 (0.54–0.81)	1.55	0.88	-11.6% (-21.0%, -1.0%) *P = 0*.*04*	0.57 (0.50–0.65)
	***(b) By continent***												
	North America	0.33	0.18	-10.1% (-27.6%, 11.5%) *P = 0*.*2*	0.53 (0.46–0.62)	0.11	0.06	-8.0% (-23.3%, 10.4%) *P = 0*.*2*	0.59 (0.44–0.79)	2.16	1.10	-10.9% (-29.7%, 12.8%) *P = 0*.*2*	0.51 (0.43–0.61)
	Europe	0.16	0.09	-15.6% (-25.5%, -4.4%) *P = 0*.*02*	0.54 (0.44–0.66)	0.06	0.03	-13.4% (-21.1%, -4.8%) *P = 0*.*02*	0.59 (0.40–0.88)	1.04	0.54	-16.5% (-27.5%, -3.9%) *P = 0*.*03*	0.52 (0.41–0.65)
	Oceania^c^	0.24	0.22	-4.0% (-15.4%, 9.0%) *P = 0*.*4*	0.96 (0.74–1.24)	0.08	0.08	-3.6% (-17.5%, 12.6%) *P = 0*.*5*	0.98 (0.60–1.60)	1.47	1.39	-4.2% (-15.2%, 8.3%) *P = 0*.*3*	0.94 (0.70–1.28)
	***(c) By country***												
	Canada	0.49	0.21	-11.7% (-35.8%, 21.2%) *P = 0*.*3*	0.43 (0.35–0.51)	0.14	0.06	-8.4% (-37.4%, 33.9%) *P = 0*.*5*	0.45 (0.31–0.67)	3.37	1.41	-12.9% (-35.3%, 17.4%) *P = 0*.*2*	0.42 (0.34–0.52)
	USA	0.17	0.14	-4.7% (-7.7%, -1.6%) *P = 0*.*02*	0.83 (0.64–1.07)	0.07	0.06	-3.0% (-21.0%, 19.0%) *P = 0*.*7*	0.87 (0.56–1.35)	0.97	0.78	-5.5% (-12.8%, 2.5%) *P = 0*.*1*	0.80 (0.58–1.11)
	Denmark	0.16	0.06	-24.3% (-43.4%, 1.3%) *P = 0*.*1*	0.36 (0.19–0.67)	0.04	0.03	-14.9% (-42.9%, 26.8%) *P = 0*.*3*	0.84 (0.24–2.91)	1.13	0.27	-28.2% (-48.5%, 0.2%) *P = 0*.*1*	0.24 (0.11–0.50)
	France	0.40	0.06	-39.5% (-53.5%, -21.3%) *P = 0*.*01*	0.16 (0.08–0.29)	0.11	0.02	-36.4% (-52.2%, -15.3%) *P = 0*.*02*	0.19 (0.06–0.64)	2.74	0.39	-40.8% (-54.4%, -23.0%) *P = 0*.*01*	0.14 (0.07–0.29)
	The Netherlands	0.12	0.05	-21.8% (-32.5%, -9.3%) *P = 0*.*01*	0.44 (0.28–0.71)	0.05	0.01	-29.1% (-33.0%, -25.0%) *P = 0*.*0003*	0.27 (0.10–0.69)	0.66	0.36	-18.3% (-32.3%, -1.3%) *P = 0*.*04*	0.55 (0.32–0.95)
	UK	0.14	0.13	-3.0% (-11.9%, 6.7%) *P = 0*.*4*	0.91 (0.69–1.19)	0.05	0.05	1.4% (-5.7%, 9.0%) *P = 0*.*6*	0.99 (0.58–1.69)	0.87	0.75	-5.1% (-16.6%, 7.9%) *P = 0*.*3*	0.86 (0.63–1.19)
	Australia	0.24	0.22	-4.0% (-15.4%, 9.0%) *P = 0*.*4*	0.96 (0.74–1.24)	0.08	0.08	-3.6% (-17.5%, 12.6%) *P = 0*.*5*	0.98 (0.60–1.60)	1.47	1.39	-4.2% (-15.2%, 8.3%) *P = 0*.*3*	0.94 (0.70–1.28)
***Female***	***(a) Overall (7 countries including Canada*, *USA*, *4 European countries and Australia)***												
	Overall	0.16	0.10	-9.9% (-17.6%, -1.4%) *P = 0*.*03*	0.63 (0.56–0.71)	0.06	0.04	-9.7% (-17.0%, -1.8%) *P = 0*.*03*	0.61 (0.47–0.78)	0.96	0.61	-9.9% (-18.9%, 0.0%) *P = 0*.*05*	0.64 (0.56–0.73)
	***(b) By continent***												
	North America	0.22	0.11	-11.3% (-24.9%, 4.8%) *P = 0*.*1*	0.52 (0.44–0.62)	0.09	0.05	-10.6% (-27.6%, 10.4%) *P = 0*.*2*	0.50 (0.36–0.70)	1.27	0.67	-11.6% (-25.8%, 5.2%) *P = 0*.*1*	0.53 (0.43–0.64)
	Europe	0.10	0.07	-10.7% (-19.6%, -0.8%) *P = 0*.*04*	0.71 (0.57–0.88)	0.03	0.02	-13.4% (-28.7%, 5.2%) *P = 0*.*1*	0.71 (0.43–1.16)	0.68	0.49	-9.7% (-16.6%, -2.2%) *P = 0*.*03*	0.72 (0.57–0.90)
	Oceania^c^	0.15	0.13	-5.2% (-22.4%, 15.7%) *P = 0*.*5*	0.86 (0.63–1.17)	0.04	0.04	-5.3% (-27.5%, 23.7%) *P = 0*.*6*	0.86 (0.42–1.75)	1.00	0.86	-5.2% (-21.7%, 14.8%) *P = 0*.*4*	0.86 (0.62–1.20)
	***(c) By country***												
	Canada	0.31	0.15	-10.8% (-29.0%, 12.1%) *P = 0*.*2*	0.49 (0.39–0.61)	0.13	0.06	-9.7% (-32.9%, 21.4%) *P = 0*.*4*	0.48 (0.32–0.72)	1.76	0.87	-11.5% (-30.3%, 12.4%) *P = 0*.*2*	0.50 (0.39–0.63)
	USA	0.14	0.08	-12.4% (-18.3%, -6.2%) *P = 0*.*009*	0.57 (0.42–0.76)	0.06	0.03	-12.1% (-16.1%, -8.0%) *P = 0*.*003*	0.57 (0.33–0.99)	0.83	0.47	-12.6% (-21.4%, -2.8%) *P = 0*.*03*	0.57 (0.40–0.79)
	Denmark	0.11	0.10	-5.6% (-25.8%, 20.1%) *P = 0*.*5*	0.93 (0.54–1.60)	0.04	0.03	-12.6% (-42.3%, 32.5%) *P = 0*.*4*	0.74 (0.23–2.36)	0.66	0.67	-2.6% (-23.4%, 23.8%) *P = 0*.*8*	1.02 (0.56–1.87)
	France	0.26	0.07	-33.9% (-49.9%, -12.9%) *P = 0*.*02*	0.27 (0.14–0.50)	0.10	0.04	-29.0% (-55.7%, 13.6%) *P = 0*.*1*	0.39 (0.12–1.22)	1.55	0.32	-36.4% (-45.6%, -25.6%) *P = 0*.*003*	0.21 (0.10–0.43)
	The Netherlands	0.11	0.04	-22.8% (-32.9%, -11.1%) *P = 0*.*01*	0.39 (0.24–0.61)	0.03	0.01	-19.9% (-45.5%, 17.5%) *P = 0*.*2*	0.41 (0.14–1.23)	0.75	0.28	-23.7% (-30.9%, -15.7%) *P = 0*.*003*	0.38 (0.23–0.63)
	UK	0.07	0.09	1.0% (-20.5%, 28.3%) *P = 0*.*9*	1.36 (0.98–1.87)	0.02	0.03	-4.0% (-33.1%, 37.7%) *P = 0*.*7*	1.51 (0.68–3.37)	0.46	0.61	2.4% (-16.6%, 25.7%) *P = 0*.*7*	1.31 (0.94–1.83)
	Australia	0.15	0.13	-5.2% (-22.4%, 15.7%) *P = 0*.*5*	0.86 (0.63–1.17)	0.04	0.04	-5.3% (-27.5%, 23.7%) *P = 0*.*6*	0.86 (0.42–1.75)	1.00	0.86	-5.2% (-21.7%, 14.8%) *P = 0*.*4*	0.86 (0.62–1.20)

ASR–age standardised rate; SRR–standardised rate ratio (compared to 1988–1992); 5-year AvPC–average percent change between successive 5-year periods.

^a^ Incidence rates were age-standardised using the Segi 1960 World Standard Population.

^b^ 5-year average percent change in the standardised incidence rates was estimated over the period 1988–1992, 1993–1997, 1998–2002, 2003–2007 and 2008–2012. Negative signs indicate decrease in the age-standardised rates over time.

^c^ Oceania includes Australia only.

Note) Data are only included in the current analysis from cancer registries that reported for the entire period 1988–2012 and that also satisfied a priori conditions (see Materials and methods). Therefore, the age-standardised incidence rate of each country reported in the above table does not necessarily correspond to each country’s national statistics.

Details of the SRRs for each 5-year period compared to 1988–1992 are found in S3–S5 Tables.

## Discussion

### Brief summary of the main results

We found that the relative proportion of anal cancer that is SCC tended to be lower in men than in women, and lower in those aged 60+ years than in those aged <60 years (and vice versa for anal ADC). We also found that the incidence of SCC more than doubled between 1988–1992 and 2008–2012, in both men and women, when pooling data from seven high-income countries in North America, Europe and Oceania. In both men and women these increases were highest in those aged <60 years (134% and 176%, respectively). By contrast, there were significant decreases in the incidence of ADC in men (40%) and women (37%), with similar decreases in those aged <60 and 60+ years. The result of these competing trends was a significant increase in the overall incidence of anal cancer in men and women of all ages since 1988–1992 (overall increase 35% in men and 75% in women; increase in consecutive 5-year periods of 8% and 16%, respectively).

### Potential explanation for the findings

Our findings are generally consistent with previous studies, which have reported trends in the incidence of anal cancer in the general population in the specific countries included in this analysis. Consistent with previous studies, in our study the relative proportion of SCC was lower in men than in women, and conversely the relative proportion of ADC was higher in men than in women over the period considered. [14, 17, 20, 21] We found significant increases in the incidence of SCC in both men and women across all ages in line with findings from previous studies reporting on Canada [11], the US [12–14], Denmark [17], South East England [19], Scotland [20], and Australia [21]. In our study, although the pooled analysis showed significant decrease in the incidence of ADC in men and women across all ages, the ADC incidence in around one third of individual countries appeared somewhat stable possibly due to small sample size. Our results are consistent with, and complement, a recent publication by Islami *et al*. that described trends in anal cancer incidence across all ages in 18 countries using the IARC’s *CI5* data. [22] Consistent with our findings, Islami *et al*. reported that the incidence of SCC increased in both men and women in the seven countries common to those included in our analysis, whereas the incidence of ADC decreased or was stable in most populations studied. However, we have applied *a priori* quality conditions to determine which cancer registries would be included in our study, which allowed pooled estimates of the changes in the anal cancer incidence rates across the countries over the same period to be done, thereby reducing statistical uncertainty due to small sample size (an issue given that anal cancer is a rare disease at the population level). We have also included additional 5 years of data, up to 2012. Therefore, overall our findings are consistent with the prior literature, but we have extended the prior findings by updating the analysis, providing more detail on the age-specific trends, and pooling across regions. We have also strengthened the findings by applying more stringent requirements on the quality and representativeness of the data from included registries.

The main risk factors for anal cancer, and for SCC in particular, are those related to infection with HPV (for example multiple sexual partners and receptive anal intercourse), immune deficiency (i.e. HIV-positive and organ transplant recipients) and smoking. [6, 31–37] HPV is the most important aetiological agent for anal cancer [38] and HPV DNA has been detected in more than 80% of anal cancer cases and more than 90% of anal intraepithelial neoplasia (AIN) cases. [3, 4] SCC is strongly associated with HPV infection, with more than 90% of SCC cases positive for high risk HPV types (HPV 16 and 18 in particular); whereas anal ADC behaves more like low-rectal carcinoma, with about 40% of cases being HPV-positive. [1, 39] High risk groups for anal cancer include men who have sex with men (MSM) [36], women with a history of other HPV-related lower genital tract disease (i.e. cervical intraepithelial neoplasia grade 3, cervical cancer, vulvar high-grade squamous intraepithelial lesion or vulvar cancer) [35] and people with HIV infection or a history of organ transplantation. [37] A meta-analysis reported that the standardised incidence ratio of non-AIDS cancers among HIV-infected individuals compared to general population rates was 28 [40] and the risk of anal cancer in HIV-positive MSM was as high as 131 per 100,000. [41]

We found marked increases in recent trends in the incidence of SCC of the anus in men and women, both aged <60 and 60+, but particularly in those aged <60 years, where the incidence rate has at least doubled over the period under examination. This is in line with previous studies of anal cancer, [14, 17, 19] and with our previously reported results that the age-standardised incidence of vulvar cancer in women aged <60 years has significantly increased in developed countries. [23, 24] Increases in both anal and vulvar cancer are consistent with changing sexual behaviours (e.g. age at sexual debut and the number of sexual partners) and thus increasing levels of HPV exposure in cohorts born around/after 1950. The attributable fraction of HPV in anal cancer is greater than that in vulvar cancer, and this could explain why the findings from the current paper show stronger increases than were observed for vulvar cancer. [23, 24] Changes in anal cancer may also reflect increased exposure to HPV over time due to other population changes in sexual behaviour, such as an increase in the number of individuals engaging in receptive anal intercourse in both heterosexual and homosexual relationships. The increasing trends in the incidence of AIN and in situ SCC [12, 13, 17] also correspond well to the increasing levels of HPV exposure and significant increases in the incidence of SCC of the anus. We used 60 years of age as a surrogate measure of changes in sexual behaviour, but our results were unchanged when we used age 50 years as a surrogate. Data on HPV prevalence in older birth cohorts is very limited, making direct comparison of HPV prevalence over time impossible, however sexual behaviour surveys provide some insight. National surveys of sexual behaviour in Australia [42] France [43] the USA and the UK have also reported an increasing prevalence of heterosexual anal intercourse among both men and women born after around 1950 compared to those born earlier, although it is unclear whether the increase reflects a true increase in the prevalence or increased willingness by respondents to report sensitive sexual behaviour.

Some studies have suggested possible alternative mechanisms for anal HPV infection other than anal intercourse. Among heterosexual men, prior genital HPV infection was associated with a higher risk of a subsequent type-specific anal infection, possibly due to autoinoculation, and the risk was independent of having sexual intercourse with female partners. [44] A large cohort study of healthy women found that about 80% of women with both anal and cervical infections shared at least one HPV type. In that study, anal intercourse was associated with anal infection only among those without a concomitant cervical infection, and it was not associated with anal HPV infection among those with anal and cervical coinfection. [45] Post-toilet wiping behaviours have also been linked with the prevalence of anal HPV infection among women. In a cross-sectional study, front-to-back wiping was associated with significantly increased prevalence of anal cytological/histological abnormalities, infection with high risk HPV types and high risk HPV co-carriage between gynaecological and anal samples, whereas dabbing post-toilet was significantly associated with decreased prevalence of the abnormalities and high risk HPV infection. [46] These studies suggest that these other anatomical sites act as potential reservoirs of infection for each other, and therefore the increase in anal infections may reflect an increase in HPV infections generally, rather than solely in the anal canal.

It is also possible that the increase we have observed in the incidence of anal cancer could be partially due to an increase in the number of people infected with HIV. However, this group of people comprise a relatively small proportion of the general population in these countries and the contribution of HIV infection to the anal cancer incidence on a population level is unclear. Earlier studies reported that highly active antiretroviral therapy (HAART), which was widely introduced in 1996, has been associated with an increase in the incidence of anal cancer among individuals with HIV infection, particularly among men and women aged 35–54 via partial immune restoration and thus improved survival. [15, 47] However, early initiation of antiretroviral therapy could contribute to lowering the risk of cancers in HIV-infected population as seen in recent studies. [48, 49] Due to data limitations, we could not examine to what extent the increases in SCC of the anus were from high risk groups with respect to behaviour or HIV status. A data linkage study in the USA found that the incidence of anal cancer among people with HIV increased steeply during 1996 to 2000, reached a plateau during 2001 to 2008 and declined during 2008 to 2012. [50] However, in our analysis, the incidence of SCC of the anus continued to increase from 2003–2007 to 2008–2012 in men and women of all age groups, with the largest increase in men and women aged <60 years (from 109% to 134% and from 132% to 176%, respectively), which resulted in increase in the overall anal cancer incidence (from 27% to 35% in men of all ages; from 47% to 75% in women of all ages). Therefore, although the current study could not adjust for the HIV status, it is less likely that the increase in the number of HIV-infected people played a significant role in the increased anal cancer incidence.

In our study, the incidence of ADC of the anus decreased in men and women of all age groups across the seven countries, although significant decreases were not observed in each of the individual countries, probably due to small sample size. Differing trends in the incidence rates of anal cancer by histological subtypes may be explained by different risk factor profiles. However, most of the known risk factors of anal cancer are strongly linked to SCC and little is known about ADC. Epidemiological studies suggest that ADC of the anus is much less closely related to HPV infection than SCC. [51] Since the rectum is made up of glandular cells and not squamous cells, it has been speculated that many cases of ADC of the anus may have actually arisen from the rectum. Similarly, SCC of the rectum, although rare, is likely to be anal cancer that has been miscoded to the rectum or overlapping anal lesions. [51] It is not known what impact, if any, potential miscoding may have had on the trends we identified here.

In our analysis, although formal statistical analyses comparing the differences in the SRRs between men and women were not performed, the magnitude of the SRRs (2008–2012 vs. 1988–1992), considering anal cancer overall and SCC and ADC separately, seemed to be generally comparable between men and women in each age group. A few studies have reported that the standardised incidence rates of anal cancer were higher in women but that the rate of increase in the incidence of anal cancer in the last few decades, either combining all histological subtypes or SCC separately, was higher in men than in women. [12, 21] However, the different findings could be partially explained by either the greater rate of increase in *in situ* cases (included in some earlier studies, but not here) in men than women or using the different population for standardisation.^12, 21^

Given that the age-specific incidence of anal cancer is greater in older age groups and that the findings of this study suggest increased SCC incidence in men and women from more recent birth cohorts, the rate of anal cancer could be expected to further increase in the future, and case numbers would additionally be expected to increase substantially due to population growth. The prophylactic HPV vaccine is likely to counter these effects to some extent in the longer term, depending on the vaccination program (targeting female only vs. both male and female, vaccination coverage, and the degree of herd immunity induced). However, a substantial reduction in anal cancer cases is unlikely within the next few decades because in both men and women incidence rates are higher in older age groups, who will not contain cohorts offered vaccination for some time. In addition, the burden of anal cancer in men is largely concentrated in the MSM population, who are less likely to benefit from herd immunity induced by HPV vaccination in females than heterosexual men [52], although some settings offer targeted HPV vaccination for MSM. While some countries now offer HPV vaccination to males as well as females, this typically started later than routine vaccination targeting females. Routine screening for anal cancer in the general population is currently not recommended due to its low prevalence. However routine screening of selected populations has been shown to be more promising, including digital anorectal exam, HPV testing and high-resolution anoscopy; however there is no consensus on the target population and modality for screening and there is minimal clinical evidence demonstrating the efficacy of anal cancer screening. [53] A few screening trials targeting high risk populations (i.e. HIV-positive men and women) are underway. [54, 55] In the meantime, HPV testing and partial genotyping of anal samples might be of benefit to some extent to select pre-cancerous/cancerous lesions associated with HPV 16/18. [53]

In conclusion, this study systematically explored trends in anal cancer incidence by sex, age group and histological subtype across multiple countries using data from cancer registries where data collection for anal cancer was reliable over 25 years. The significant increases in the incidence of SCC of the anus in both men and women are consistent with changing sexual behaviours and increasing exposure to HPV.

## Supporting information

S1 Fig Pooled age-specific incidence of squamous cell carcinoma of the anus by birth cohort in men and women born from 1900 to 1983, for each of the five 5-yearly average rates (1988–92, 1993–97, 1998–2002, 2003–2007, 2008–2012). (DOCX)

S2 Fig Pooled age-specific incidence of adenocarcinoma of the anus by birth cohort in men and women born from 1900 to 1983, for each of the five 5-yearly average rates (1988–92, 1993–97, 1998–2002, 2003–2007, 2008–2012). (DOCX)

S1 Table Cancer registries included in the analysis. (DOCX)

S2 Table Number of incident anal cancer cases and size of male and female population at risk in the cancer registries included in the analysis. (DOCX)

S3 Table Standardised rate ratios in the age-standardised anal cancer incidence rates (per 100,000 individuals), compared to 1988–1992, in selected high income countries: All histological types. (DOCX)

S4 Table Standardised rate ratios in the age-standardised anal cancer incidence rates (per 100,000 individuals), compared to 1988–1992, in selected high income countries: Squamous cell carcinoma of the anus. (DOCX)

S5 Table Standardised rate ratios in the age-standardised anal cancer incidence rates (per 100,000 individuals), compared to 1988–1992, in selected high income countries: Adenocarcinoma of the anus. (DOCX)
