# Correction: Anal cancer in high-income countries: Increasing burden of disease

**DOI:** 10.1371/journal.pone.0216884

**Published:** 2019-05-08

**Authors:** Yoon-Jung Kang, Megan Smith, Karen Canfell

Fig 1 contains errors. In Fig 1, the panel showing the pooled age-specific incidence rates of adenocarcinoma (ADC) in males is duplicated. One of the duplicates (i.e. middle panel on the right) should have shown the pooled age-specific incidence rates of squamous cell carcinoma (SCC) in females. Please see the correct Fig 1 here.

**Fig 1:**
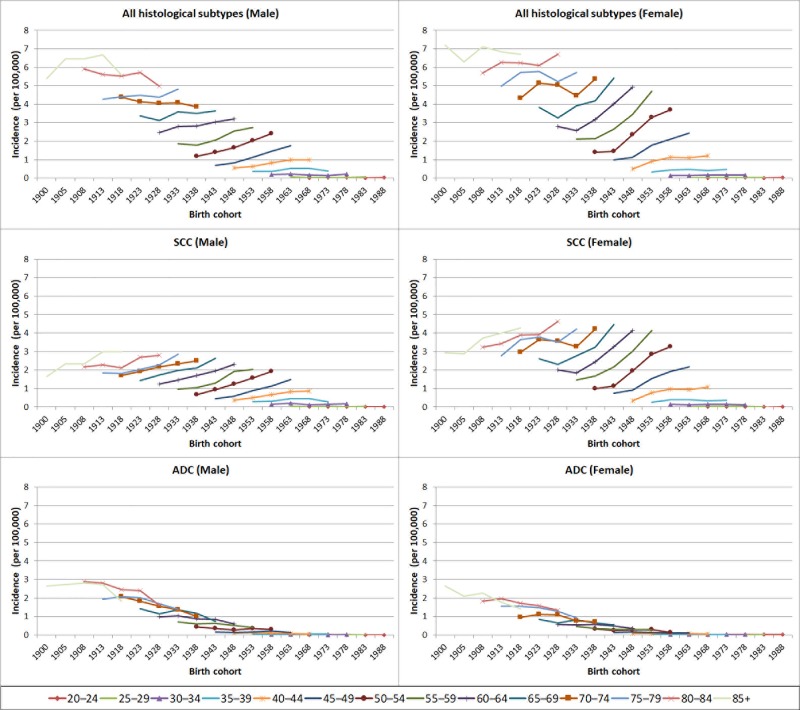
Pooled age-specific anal cancer incidence rates by birth cohort in men and women born from 1900 to 1983, for each of the five 5-yearly average rates (1988–92, 1993–97, 1998–2002, 2003–2007, 2008–2012). SCC–squamous cell carcinoma; ADC–adenocarcinoma; M–male; F- female.

There are errors in Fig 2. Fig 2 illustrates the age-standardised incidence rates of anal cancer by histological subtype, sex and age groups in four individual panels–one each for the overall rates across the seven countries (i.e. overall), North America, Europe, and Oceania. In Fig 2, the age-standardised incidence rates illustrated in North America (i.e. top panel on the right), Europe (i.e. bottom panel on the left) and Oceania (i.e. bottom panel on the right) are identical to the overall rates (i.e. top panel on the left). Each panel should have shown continent-specific rates. Please see the correct Fig 2 here.

**Fig 2:**
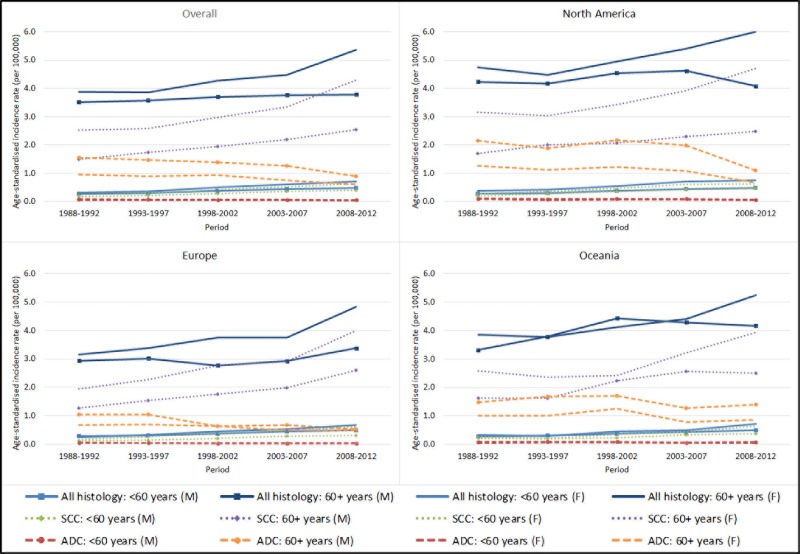
Pooled anal cancer incidence rates in selected high income countries, by region, sex, histological subtype and age group. Note) Oceania includes Australia only. SCC–squamous cell carcinoma; ADC–adenocarcinoma; M–male; F- female.

S2 Fig is also incorrect. S2 Fig consists of six individual panels of pooled age-specific incidence rates of adenocarcinoma (ADC) by continent. In S2 Fig, incidence rates of squamous cell carcinoma (SCC) in females in North America (i.e. top panel on the right) is included instead of ADC in females in North America. Please see the correct S2 Fig here.

These errors do not affect the study’s findings, nor the conclusions drawn.

## Supporting information

S2 Fig Pooled age-specific incidence of adenocarcinoma of the anus by birth cohort in men and women born from 1900 to 1983, for each of the five 5-yearly average rates (1988–92, 1993–97, 1998–2002, 2003–2007, 2008–2012). (DOCX)
