# Supplementary material for: Anal cancer in high-income countries: Increasing burden of disease
Source: PLoS One. 2018 Oct 19;13(10):e0205105. doi: 10.1371/journal.pone.0205105 (PMC6195278; doi:10.1371/journal.pone.0205105)
Supplement: S1 Table — (DOCX) [file pone.0205105.s003.docx]

S1 Table. Cancer registries included in the analysis

| **Country^a^** | **Cancer registries included in the analysis** | **Registry coverage** | **Population coverage^b^** |
| --- | --- | --- | --- |
| Canada | The Alberta Cancer Registry, the British Columbia Cancer Registry, the Manitoba Cancer Registry, the New Brunswick Provincial Cancer Registry, the Newfoundland and Labrador Provincial Tumour Registry, the Northwest Territories Cancer Registry, the Nova Scotia Cancer Registry, the Ontario Cancer Registry, the Prince Edward Island Cancer Registry, the Saskatchewan Cancer Registry | Each registry covers the entire province | 78% |
| USA | Participant registries in the Surveillance, Epidemiology, and End Results (SEER) Program: five states (Connecticut, Iowa, New Mexico, Utah, and Hawaii) and four metropolitan areas (the San Francisco Bay area, California; Detroit, Michigan; Atlanta, Georgia; and Seattle, Washington). | State/metropolitan areas as specified | 10% |
| Denmark | The Danish Cancer Registry | National cancer registry | 100% |
| France | The Bas-Rhin Cancer Registry, Calvados, the Doubs Cancer Registry, the Haut-Rhin Cancer Registry, the Hérault Cancer Registry, the Isère Cancer Registry, the Somme Cancer Registry, the Tarn Cancer Registry | Each registry covers the entire department/region | 6% |
| The Netherlands | The Netherlands Cancer Registry | National cancer registry | 100% |
| UK | The North Western Regional Cancer Registry, the South West office of the National Cancer Registration Service, the South West office of the National Cancer Registration Service, the West Midlands Cancer Intelligence Unit (WMCIU), the Scottish Cancer Registry | Each registry covers the entire region | 38% |
| Australia | The New South Wales Central Cancer Registry, the South Australian Cancer Registry, The Tasmanian Cancer Registry, the Victorian Cancer Registry, the Western Australian Cancer Registry | Each registry covers the entire state | 80% |

^a^ Countries were included in the analysis if the available registry data fulfilled several *a priori* conditions: i) at least one jurisdictional cancer registry in the country covered its entire catchment area and reported for the whole period from 1988 to 2012; ii) information on histological subtype was available; iii) the population at risk in each 5-year age group was available; iv) the reported incidence of SCC and ADC of the anus for the first and the last 5-year period (i.e. 1988-1992 and 2008-2012) was not zero (as this may have potentially indicated under-reporting associated with potential disease misclassification); and v) no obvious signs of over-reporting associated with potential disease misclassification (e.g. age-standardised incidence rate of anal cancer ≥ 5 per 100,000, which may have included ADC arising from the rectum).

^b^ For each country, the population coverage was calculated by dividing the sum of the populations at risk in each of the included registries (average over 2008-2012) recorded in Cancer Incidence in Five Continents Volume 11 by the United Nations female population estimate in 2010 for the entire country (using the medium variant for fertility, migration and mortality rates).
