# Supplementary material for: Anal cancer in high-income countries: Increasing burden of disease
Source: PLoS One. 2018 Oct 19;13(10):e0205105. doi: 10.1371/journal.pone.0205105 (PMC6195278; doi:10.1371/journal.pone.0205105)
Supplement: S2 Table — (DOCX) [file pone.0205105.s004.docx]

S2 Table. Number of incident anal cancer cases and size of male and female population at risk in the cancer registries included in the analysis

|  | | **Anal cancer cases diagnosed in each period** | | | | |  | **Population at risk in the same registry catchment areas** | | | | | |
| --- | --- | --- | --- | --- | --- | --- | --- | --- | --- | --- | --- | --- | --- |
| **Country** | | **1988-1992** | **1993-1997** | **1998-2002** | **2003-2007** | **2008-2012** | **1988-1992** | | **1993-1997** | **1998-2002** | **2003-2007** | **2008-2012** |  |
| ***(a) Male*** |  | | | | | | | | | | |  |  |
| Canada | | 551 | 613 | 778 | 917 | 874 | 51,567,693 | | 55,048,270 | 57,892,386 | 61,025,390 | 64,455,805 |  |
| USA | | 420 | 560 | 738 | 911 | 1,094 | 52,696,554 | | 61,313,183 | 65,894,745 | 68,959,361 | 71,473,283 |  |
| Denmark | | 115 | 133 | 137 | 157 | 192 | 12,678,700 | | 12,925,220 | 13,189,753 | 13,411,122 | 13,735,532 |  |
| France | | 101 | 110 | 88 | 113 | 92 | 10,753,148 | | 11,123,432 | 11,480,065 | 11,919,096 | 9,263,204 |  |
| The Netherlands | | 133 | 152 | 221 | 293 | 409 | 29,684,100 | | 38,210,130 | 39,398,984 | 40,341,582 | 41,095,905 |  |
| UK | | 376 | 512 | 525 | 683 | 763 | 43,027,686 | | 51,489,485 | 51,484,653 | 58,315,528 | 56,601,078 |  |
| Australia | | 266 | 323 | 414 | 489 | 584 | 34,105,749 | | 35,610,096 | 37,469,298 | 39,387,832 | 42,272,084 |  |
|  |  | | | | | | | | | | |  |  |
| ***(b) Female*** |  | | | | | | | | | | |  |  |
| Canada | | 659 | 781 | 1,011 | 1,235 | 1,447 | 52,208,108 | | 55,921,380 | 58,925,418 | 62,078,324 | 65,685,400 |  |
| USA | | 712 | 807 | 1,031 | 1,304 | 1,605 | 54,942,117 | | 63,890,849 | 67,930,988 | 70,686,135 | 73,606,406 |  |
| Denmark | | 175 | 258 | 305 | 332 | 423 | 13,047,100 | | 13,263,467 | 13,497,342 | 13,689,087 | 13,970,691 |  |
| France | | 213 | 233 | 265 | 270 | 254 | 11,359,489 | | 11,742,527 | 12,156,219 | 12,602,372 | 9,764,393 |  |
| The Netherlands | | 157 | 243 | 306 | 374 | 497 | 30,370,100 | | 39,063,226 | 40,240,534 | 41,213,163 | 41,943,455 |  |
| UK | | 489 | 733 | 905 | 1,089 | 1,380 | 45,191,010 | | 53,658,698 | 54,319,468 | 61,151,444 | 58,989,298 |  |
| Australia | | 375 | 400 | 523 | 598 | 856 | 34,403,062 | | 36,073,619 | 38,065,626 | 40,064,040 | 42,796,314 |  |

Note) Data are only included in the current analysis from cancer registries that reported for the entire period 1988-2007 and that also satisfied *a priori* conditions (see Materials and Methods).
