# Supplementary material for: Anal cancer in high-income countries: Increasing burden of disease
Source: PLoS One. 2018 Oct 19;13(10):e0205105. doi: 10.1371/journal.pone.0205105 (PMC6195278; doi:10.1371/journal.pone.0205105)
Supplement: S3 Table — (DOCX) [file pone.0205105.s005.docx]

S3 Table. Standardised rate ratios in the age-standardised anal cancer incidence rates (per 100,000 individuals), compared to 1988-1992, in selected high income countries: all histological types

|  |  | **Standardised rate ratios (95% CI)** | | | | | | | | | | | |
| --- | --- | --- | --- | --- | --- | --- | --- | --- | --- | --- | --- | --- | --- |
|  | **Continent/** | **All ages** | | | | **<60 years** | | | | **60+ years** | | | |
| **Sex** | **Country** | **1993-1997** | **1998-2002** | **2003-2007** | **2008-2012** | **1993-1997** | **1998-2002** | **2003-2007** | **2008-2012** | **1993-1997** | **1998-2002** | **2003-2007** | **2008-2012** |
| **Male** | ***(a) Overall (7 countries including Canada, USA, 4 European countries and Australia)*** | | | | | | | | | | | | |
|  | Overall | 1.04  (0.98-1.11) | 1.16  (1.09-1.23) | 1.27  (1.21-1.35) | 1.35  1.28-1.42) | 1.08  (0.98-1.20) | 1.33  (1.21-1.46) | 1.59  (1.46-1.73) | 1.77  (1.62-1.92) | 1.02  (0.94-1.10) | 1.05  (0.98-1.13) | 1.07  (1.00-1.15) | 1.08  (1.00-1.15) |
|  | ***(b) By continent*** | | | | | | | | | | | | |
|  | North America | 1.06  (0.97-1.15) | 1.22  (1.12-1.32) | 1.32  (1.22-1.42) | 1.28  (1.18-1.38) | 1.17  (1.01-1.34) | 1.44  (1.27-1.64) | 1.67  (1.48-1.88) | 1.77  (1.57-1.98) | 0.99  (0.88-1.10) | 1.07  (0.96-1.19) | 1.09  (0.98-1.21) | 0.97  (0.87-1.07) |
|  | Europe | 1.03  (0.93-1.14) | 1.06  (0.96-1.16) | 1.20  (1.10-1.32) | 1.39  (1.27-1.51) | 1.03  (0.86-1.23) | 1.25  (1.06-1.47) | 1.55  (1.33-1.81) | 1.78  (1.54-2.07) | 1.03  (0.91-1.16) | 0.94  (0.83-1.07) | 1.00  (0.89-1.12) | 1.15  (1.03-1.29) |
|  | Oceania^a^ | 1.07  (0.90-1.26) | 1.23  (1.06-1.44) | 1.32  (1.14-1.53) | 1.41  (1.22-1.63) | 0.96  (0.73-1.26) | 1.09  (0.85-1.41) | 1.35  (1.07-1.71) | 1.62  (1.30-2.02) | 1.14  (0.93-1.41) | 1.34  (1.10-1.63) | 1.29  (1.07-1.57) | 1.26  (1.04-1.51) |
|  | ***(c) By country*** | | | | | | | | | | | | |
|  | Canada | 1.01  (0.90-1.14) | 1.13  (1.01-1.26) | 1.19  (1.07-1.32) | 0.99  (0.89-1.11) | 1.22  (0.98-1.50) | 1.42  (1.17-1.73) | 1.63  (1.36-1.96) | 1.47  (1.23-1.77) | 0.92  (0.80-1.07) | 1.00  (0.87-1.15) | 0.98  (0.86-1.13) | 0.78  (0.68-0.90) |
|  | USA | 1.11  (0.98-1.26) | 1.32  (1.17-1.49) | 1.47  (1.31-1.64) | 1.62  (1.45-1.80) | 1.13  (0.93-1.36) | 1.45  (1.23-1.71) | 1.69  (1.45-1.98) | 1.99  (1.72-2.31) | 1.10  (0.92-1.31) | 1.19  (1.00-1.42) | 1.25  (1.06-1.48) | 1.27  (1.08-1.48) |
|  | Denmark | 1.21  (0.93-1.57) | 1.20  (0.93-1.56) | 1.36  (1.06-1.74) | 1.54  (1.22-1.96) | 1.78  (1.13-2.79) | 1.95  (1.27-2.99) | 2.11  (1.39-3.22) | 2.29  (1.51-3.47) | 0.93  (0.68-1.28) | 0.84  (0.61-1.16) | 0.99  (0.72-1.34) | 1.18  (0.89-1.56) |
|  | France | 0.97  (0.74-1.29) | 0.75  (0.56-1.01) | 0.87  (0.66-1.16) | 0.86  (0.64-1.16) | 1.02  (0.60-1.72) | 1.08  (0.66-1.78) | 1.37  (0.86-2.16) | 1.31  (0.80-2.14) | 0.95  (0.69-1.32) | 0.59  (0.41-0.85) | 0.63  (0.44-0.91) | 0.64  (0.44-0.93) |
|  | The Netherlands | 0.84  (0.66-1.07) | 1.15  (0.93-1.43) | 1.38  (1.13-1.69) | 1.77  (1.48-2.13) | 0.71  (0.48-1.05) | 1.17  (0.84-1.62) | 1.46  (1.08-1.98) | 1.80  (1.36-2.39) | 0.95  (0.70-1.29) | 1.14  (0.86-1.52) | 1.32  (1.01-1.73) | 1.75  (1.38-2.22) |
|  | UK | 1.11  (0.96-1.27) | 1.09  (0.95-1.25) | 1.21  (1.07-1.38) | 1.37  (1.21-1.55) | 1.06  (0.82-1.36) | 1.20  (0.94-1.52) | 1.55  (1.25-1.92) | 1.81  (1.47-2.23) | 1.13  (0.96-1.33) | 1.03  (0.88-1.22) | 1.01  (0.87-1.19) | 1.12  (0.96-1.30) |
|  | Australia | 1.07  (0.90-1.26) | 1.23  (1.06-1.44) | 1.32  (1.14-1.53) | 1.41  (1.22-1.63) | 0.96  (0.73-1.26) | 1.09  (0.85-1.41) | 1.35  (1.07-1.71) | 1.62  (1.30-2.02) | 1.14  (0.93-1.41) | 1.34  (1.10-1.63) | 1.29  (1.07-1.57) | 1.26  (1.04-1.51) |
| **Female** | ***(a) Overall (7 countries including Canada, USA, 4 European countries and Australia)*** | | | | | | | | | | | | |
|  | Overall | 1.06  (1.01-1.12) | 1.30  (1.23-1.37) | 1.47  (1.40-1.54) | 1.75  (1.67-1.83) | 1.17  (1.06-1.29) | 1.61  (1.48-1.75) | 1.95  (1.81-2.11) | 2.31  (2.14-2.48) | 0.99  (0.93-1.06) | 1.10  (1.03-1.17) | 1.15  (1.09-1.23) | 1.38  (1.31-1.46) |
|  | ***(b) By continent*** | | | | | | | | | | | | |
|  | North America | 1.01  (0.94-1.10) | 1.21  (1.12-1.30) | 1.42  (1.33-1.52) | 1.54  (1.44-1.65) | 1.13  (0.99-1.29) | 1.47  (1.30-1.65) | 1.86  (1.67-2.07) | 1.98  (1.78-2.19) | 0.94  (0.86-1.03) | 1.04  (0.95-1.14) | 1.14  (1.04-1.24) | 1.26  (1.16-1.37) |
|  | Europe | 1.18  (1.08-1.29) | 1.46  (1.34-1.59) | 1.58  (1.46-1.71) | 2.02  (1.88-2.18) | 1.36  (1.17-1.60) | 1.90  (1.65-2.19) | 2.23  (1.96-2.54) | 2.84  (2.51-3.21) | 1.07  (0.97-1.19) | 1.19  (1.08-1.32) | 1.19  (1.08-1.31) | 1.53  (1.40-1.68) |
|  | Oceania^a^ | 0.93  (0.80-1.08) | 1.20  (1.04-1.38) | 1.30  (1.14-1.49) | 1.70  (1.50-1.92) | 0.85  (0.65-1.13) | 1.39  (1.10-1.75) | 1.53  (1.23-1.91) | 2.18  (1.79-2.66) | 0.98  (0.82-1.17) | 1.07  (0.90-1.27) | 1.14  (0.96-1.36) | 1.36  (1.17-1.59) |
|  | ***(c) By country*** | | | | | | | | | | | | |
|  | Canada | 1.05  (0.94-1.17) | 1.24  (1.11-1.37) | 1.44  (1.31-1.59) | 1.51  (1.37-1.66) | 1.09  (0.90-1.32) | 1.33  (1.12-1.59) | 1.83  (1.57-2.14) | 1.87  (1.61-2.17) | 1.03  (0.90-1.17) | 1.17  (1.03-1.33) | 1.19  (1.05-1.35) | 1.27  (1.13-1.43) |
|  | USA | 0.98  (0.88-1.10) | 1.18  (1.06-1.30) | 1.40  (1.27-1.54) | 1.57  (1.43-1.72) | 1.17  (0.97-1.40) | 1.59  (1.35-1.87) | 1.89  (1.63-2.20) | 2.08  (1.80-2.40) | 0.87  (0.76-0.99) | 0.92  (0.81-1.05) | 1.09  (0.96-1.23) | 1.25  (1.12-1.40) |
|  | Denmark | 1.44  (1.16-1.78) | 1.79  (1.47-2.19) | 1.94  (1.59-2.35) | 2.37  (1.97-2.84) | 1.43  (1.01-2.03) | 2.30  (1.69-3.12) | 2.51  (1.86-3.39) | 2.91  (2.19-3.87) | 1.45  (1.12-1.88) | 1.39  (1.07-1.80) | 1.47  (1.14-1.89) | 1.93  (1.53-2.43) |
|  | France | 1.00  (0.81-1.23) | 1.10  (0.90-1.34) | 1.10  (0.90-1.35) | 1.33  (1.08-1.63) | 1.04  (0.71-1.54) | 1.24  (0.87-1.77) | 1.50  (1.08-2.08) | 1.76  (1.25-2.48) | 0.97  (0.77-1.23) | 1.01  (0.80-1.28) | 0.86  (0.67-1.10) | 1.05  (0.82-1.35) |
|  | The Netherlands | 1.21  (0.98-1.51) | 1.49  (1.22-1.83) | 1.78  (1.47-2.16) | 2.28  (1.91-2.72) | 1.87  (1.25-2.79) | 2.48  (1.73-3.54) | 3.44  (2.50-4.73) | 4.41  (3.28-5.92) | 0.94  (0.73-1.21) | 1.07  (0.84-1.37) | 1.08  (0.85-1.38) | 1.37  (1.10-1.72) |
|  | UK | 1.23  (1.08-1.40) | 1.58  (1.40-1.78) | 1.68  (1.50-1.88) | 2.27  (2.04-2.52) | 1.44  (1.14-1.81) | 2.02  (1.64-2.48) | 2.25  (1.86-2.73) | 3.06  (2.56-3.66) | 1.10  (0.95-1.28) | 1.31  (1.13-1.51) | 1.32  (1.16-1.52) | 1.78  (1.56-2.02) |
|  | Australia | 0.93  (0.80-1.08) | 1.20  (1.04-1.38) | 1.30  (1.14-1.49) | 1.70  (1.50-1.92) | 0.85  (0.65-1.13) | 1.39  (1.10-1.75) | 1.53  (1.23-1.91) | 2.18  (1.79-2.66) | 0.98  (0.82-1.17) | 1.07  (0.90-1.27) | 1.14  (0.96-1.36) | 1.36  (1.17-1.59) |

^a^ Oceania includes Australia only.
