# Supplementary material for: Anal cancer in high-income countries: Increasing burden of disease
Source: PLoS One. 2018 Oct 19;13(10):e0205105. doi: 10.1371/journal.pone.0205105 (PMC6195278; doi:10.1371/journal.pone.0205105)
Supplement: S4 Table — (DOCX) [file pone.0205105.s006.docx]

S4 Table. Standardised rate ratios in the age-standardised anal cancer incidence rates (per 100,000 individuals), compared to 1988-1992, in selected high income countries: squamous cell carcinoma of the anus

|  |  | **Standardised rate ratios (95% CI)** | | | | | | | | | | | |
| --- | --- | --- | --- | --- | --- | --- | --- | --- | --- | --- | --- | --- | --- |
|  | **Continent/** | **All ages** | | | | **<60 years** | | | | **60+ years** | | | |
| **Sex** | **Country** | **1993-1997** | **1998-2002** | **2003-2007** | **2008-2012** | **1993-1997** | **1998-2002** | **2003-2007** | **2008-2012** | **1993-1997** | **1998-2002** | **2003-2007** | **2008-2012** |
| **Male** | ***(a) Overall (7 countries including Canada, USA, 4 European countries and Australia)*** | | | | | | | | | | | | |
|  | Overall | 1.20  (1.10-1.31) | 1.47  (1.36-1.59) | 1.76  (1.64-1.89) | 2.00  (1.87-2.14) | 1.25  (1.10-1.41) | 1.66  (1.48-1.86) | 2.09  (1.88-2.31) | 2.34  (2.11-2.58) | 1.16  (1.03-1.30) | 1.30  (1.17-1.45) | 1.46  (1.32-1.62) | 1.69  (1.54-1.86) |
|  | ***(b) By continent*** | | | | | | | | | | | | |
|  | North America | 1.28  (1.14-1.45) | 1.49  (1.33-1.66) | 1.71  (1.54-1.90) | 1.86  (1.68-2.05) | 1.39  (1.17-1.64) | 1.77  (1.52-2.06) | 2.10  (1.82-2.41) | 2.28  (1.99-2.61) | 1.18  (1.00-1.40) | 1.21  (1.03-1.43) | 1.34  (1.15-1.57) | 1.45  (1.26-1.68) |
|  | Europe | 1.21  (1.05-1.40) | 1.54  (1.35-1.76) | 1.91  (1.69-2.15) | 2.32  (2.07-2.59) | 1.22  (0.97-1.53) | 1.74  (1.42-2.14) | 2.35  (1.95-2.81) | 2.67  (2.23-3.19) | 1.21  (1.01-1.44) | 1.39  (1.18-1.65) | 1.58  (1.35-1.85) | 2.05  (1.77-2.37) |
|  | Oceania^a^ | 0.94  (0.75-1.19) | 1.24  (1.01-1.53) | 1.59  (1.31-1.92) | 1.69  (1.41-2.03) | 0.90  (0.64-1.26) | 1.11  (0.81-1.52) | 1.60  (1.22-2.11) | 1.85  (1.43-2.40) | 0.99  (0.73-1.35) | 1.37  (1.04-1.81) | 1.57  (1.21-2.04) | 1.53  (1.19-1.97) |
|  | ***(c) By country*** | | | | | | | | | | | | |
|  | Canada | 1.38  (1.14-1.66) | 1.39  (1.16-1.68) | 1.61  (1.36-1.91) | 1.65  (1.40-1.95) | 1.55  (1.17-2.06) | 1.71  (1.31-2.23) | 2.00  (1.57-2.56) | 1.96  (1.54-2.49) | 1.24  (0.96-1.60) | 1.14  (0.88-1.47) | 1.30  (1.02-1.65) | 1.41  (1.12-1.76) |
|  | USA | 1.22  (1.05-1.42) | 1.53  (1.33-1.76) | 1.76  (1.54-2.01) | 1.98  (1.75-2.24) | 1.29  (1.04-1.59) | 1.76  (1.46-2.13) | 2.12  (1.78-2.52) | 2.44  (2.06-2.87) | 1.15  (0.91-1.44) | 1.28  (1.03-1.59) | 1.38  (1.12-1.69) | 1.49  (1.23-1.81) |
|  | Denmark | 1.17  (0.83-1.64) | 1.56  (1.14-2.13) | 1.80  (1.34-2.42) | 2.03  (1.54-2.69) | 1.68  (0.99-2.87) | 2.35  (1.46-3.78) | 2.63  (1.66-4.19) | 2.47  (1.55-3.96) | 0.84  (0.55-1.30) | 1.06  (0.70-1.61) | 1.27  (0.87-1.87) | 1.75  (1.25-2.47) |
|  | France | 1.44  (0.94-2.22) | 1.52  (1.00-2.30) | 1.88  (1.27-2.78) | 1.97  (1.31-2.96) | 1.43  (0.69-2.97) | 1.88  (0.98-3.61) | 2.67  (1.49-4.80) | 2.53  (1.34-4.80) | 1.45  (0.86-2.44) | 1.27  (0.74-2.17) | 1.34  (0.79-2.27) | 1.58  (0.93-2.67) |
|  | The Netherlands | 0.82  (0.59-1.14) | 1.53  (1.16-2.01) | 2.14  (1.68-2.73) | 2.87  (2.31-3.57) | 0.69  (0.42-1.13) | 1.39  (0.94-2.05) | 2.03  (1.44-2.87) | 2.62  (1.90-3.61) | 0.96  (0.61-1.50) | 1.69  (1.15-2.47) | 2.26  (1.61-3.19) | 3.16  (2.35-4.25) |
|  | UK | 1.43  (1.18-1.74) | 1.62  (1.34-1.95) | 1.92  (1.62-2.28) | 2.30  (1.95-2.71) | 1.43  (1.03-1.99) | 1.79  (1.32-2.44) | 2.45  (1.87-3.20) | 2.85  (2.19-3.70) | 1.43  (1.13-1.82) | 1.50  (1.18-1.89) | 1.55  (1.24-1.93) | 1.91  (1.55-2.34) |
|  | Australia | 0.94  (0.75-1.19) | 1.24  (1.01-1.53) | 1.59  (1.31-1.92) | 1.69  (1.41-2.03) | 0.90  (0.64-1.26) | 1.11  (0.81-1.52) | 1.60  (1.22-2.11) | 1.85  (1.43-2.40) | 0.99  (0.73-1.35) | 1.37  (1.04-1.81) | 1.57  (1.21-2.04) | 1.53  (1.19-1.97) |
| **Female** | ***(a) Overall (7 countries including Canada, USA, 4 European countries and Australia)*** | | | | | | | | | | | | |
|  | Overall | 1.14  (1.07-1.22) | 1.46  (1.37-1.55) | 1.75  (1.65-1.85) | 2.15  (2.04-2.26) | 1.29  (1.16-1.44) | 1.84  (1.67-2.02) | 2.32  (2.13-2.53) | 2.76  (2.54-3.00) | 1.03  (0.95-1.12) | 1.19  (1.10-1.28) | 1.33  (1.23-1.43) | 1.70  (1.59-1.82) |
|  | ***(b) By continent*** | | | | | | | | | | | | |
|  | North America | 1.11  (1.01-1.21) | 1.35  (1.24-1.47) | 1.64  (1.52-1.78) | 1.84  (1.71-1.98) | 1.32  (1.13-1.53) | 1.74  (1.52-1.99) | 2.24  (1.99-2.53) | 2.36  (2.10-2.65) | 0.96  (0.86-1.08) | 1.08  (0.97-1.21) | 1.24  (1.11-1.38) | 1.49  (1.35-1.64) |
|  | Europe | 1.31  (1.17-1.45) | 1.76  (1.59-1.94) | 2.02  (1.84-2.21) | 2.67  (2.45-2.91) | 1.49  (1.24-1.78) | 2.22  (1.89-2.60) | 2.73  (2.36-3.15) | 3.52  (3.07-4.03) | 1.17  (1.03-1.33) | 1.42  (1.26-1.61) | 1.50  (1.33-1.69) | 2.06  (1.84-2.29) |
|  | Oceania^a^ | 0.85  (0.71-1.03) | 1.10  (0.92-1.30) | 1.41  (1.20-1.66) | 1.92  (1.67-2.22) | 0.78  (0.57-1.07) | 1.28  (0.98-1.67) | 1.61  (1.26-2.05) | 2.40  (1.94-2.97) | 0.91  (0.73-1.15) | 0.94  (0.75-1.18) | 1.25  (1.01-1.54) | 1.53  (1.26-1.84) |
|  | ***(c) By country*** | | | | | | | | | | | | |
|  | Canada | 1.22  (1.06-1.41) | 1.48  (1.29-1.69) | 1.78  (1.57-2.02) | 1.91  (1.70-2.14) | 1.43  (1.13-1.79) | 1.70  (1.38-2.10) | 2.38  (1.98-2.87) | 2.35  (1.96-2.81) | 1.07  (0.89-1.28) | 1.31  (1.11-1.56) | 1.34  (1.13-1.58) | 1.58  (1.36-1.84) |
|  | USA | 1.03  (0.91-1.16) | 1.26  (1.12-1.41) | 1.55  (1.40-1.72) | 1.80  (1.63-1.98) | 1.22  (1.00-1.50) | 1.74  (1.46-2.08) | 2.12  (1.81-2.49) | 2.35  (2.02-2.75) | 0.90  (0.77-1.04) | 0.94  (0.81-1.09) | 1.18  (1.02-1.36) | 1.44  (1.26-1.63) |
|  | Denmark | 1.47  (1.16-1.87) | 1.89  (1.51-2.37) | 2.20  (1.77-2.73) | 2.64  (2.16-3.23) | 1.38  (0.94-2.02) | 2.46  (1.78-3.41) | 2.74  (2.00-3.76) | 3.12  (2.30-4.22) | 1.56  (1.15-2.12) | 1.35  (0.99-1.85) | 1.69  (1.26-2.26) | 2.19  (1.69-2.85) |
|  | France | 1.15  (0.90-1.46) | 1.42  (1.13-1.79) | 1.43  (1.14-1.80) | 1.75  (1.39-2.21) | 1.12  (0.72-1.74) | 1.57  (1.06-2.31) | 1.88  (1.31-2.69) | 2.19  (1.51-3.20) | 1.16  (0.88-1.54) | 1.32  (1.01-1.74) | 1.12  (0.84-1.49) | 1.44  (1.08-1.91) |
|  | The Netherlands | 1.49  (1.13-1.96) | 1.97  (1.53-2.53) | 2.67  (2.13-3.36) | 3.54  (2.87-4.36) | 2.41  (1.52-3.84) | 3.17  (2.08-4.82) | 4.98  (3.47-7.15) | 6.50  (4.65-9.09) | 1.03  (0.73-1.45) | 1.37  (1.00-1.88) | 1.53  (1.13-2.07) | 2.07  (1.58-2.71) |
|  | UK | 1.35  (1.15-1.59) | 1.92  (1.66-2.23) | 2.14  (1.86-2.46) | 3.07  (2.70-3.48) | 1.59  (1.22-2.08) | 2.35  (1.85-2.98) | 2.77  (2.23-3.44) | 3.87  (3.16-4.72) | 1.16  (0.96-1.42) | 1.60  (1.33-1.92) | 1.67  (1.40-1.98) | 2.47  (2.11-2.88) |
|  | Australia | 0.85  (0.71-1.03) | 1.10  (0.92-1.30) | 1.41  (1.20-1.66) | 1.92  (1.67-2.22) | 0.78  (0.57-1.07) | 1.28  (0.98-1.67) | 1.61  (1.26-2.05) | 2.40  (1.94-2.97) | 0.91  (0.73-1.15) | 0.94  (0.75-1.18) | 1.25  (1.01-1.54) | 1.53  (1.26-1.84) |

^a^ Oceania includes Australia only.
