# Supplementary material for: Anal cancer in high-income countries: Increasing burden of disease
Source: PLoS One. 2018 Oct 19;13(10):e0205105. doi: 10.1371/journal.pone.0205105 (PMC6195278; doi:10.1371/journal.pone.0205105)
Supplement: S5 Table — (DOCX) [file pone.0205105.s007.docx]

S5 Table. Standardised rate ratios in the age-standardised anal cancer incidence rates (per 100,000 individuals), compared to 1988-1992, in selected high income countries: adenocarcinoma of the anus

|  |  | **Standardised rate ratios (95% CI)** | | | | | | | | | | | |
| --- | --- | --- | --- | --- | --- | --- | --- | --- | --- | --- | --- | --- | --- |
|  | **Continent/** | **All ages** | | | | **<60 years** | | | | **60+ years** | | | |
| **Sex** | **Country** | **1993-1997** | **1998-2002** | **2003-2007** | **2008-2012** | **1993-1997** | **1998-2002** | **2003-2007** | **2008-2012** | **1993-1997** | **1998-2002** | **2003-2007** | **2008-2012** |
| **Male** | ***(a) Overall (7 countries including Canada, USA, 4 European countries and Australia)*** | | | | | | | | | | | | |
|  | Overall | 0.92  (0.84-1.02) | 0.87  (0.79-0.97) | 0.82  (0.74-0.91) | 0.60  (0.54-0.67) | 0.88  (0.72-1.07) | 0.82  (0.67-1.01) | 0.84  (0.69-1.02) | 0.66  (0.54-0.81) | 0.94  (0.84-1.06) | 0.89  (0.80-1.00) | 0.82  (0.73-0.92) | 0.57  (0.50-0.65) |
|  | ***(b) By continent*** | | | | | | | | | | | | |
|  | North America | 0.86  (0.75-0.99) | 0.96  (0.84-1.10) | 0.93  (0.81-1.06) | 0.53  (0.46-0.62) | 0.83  (0.63-1.10) | 0.86  (0.66-1.12) | 0.94  (0.73-1.21) | 0.59  (0.44-0.79) | 0.87  (0.75-1.02) | 1.00  (0.86-1.17) | 0.92  (0.79-1.07) | 0.51  (0.43-0.61) |
|  | Europe | 0.98  (0.82-1.17) | 0.62  (0.51-0.75) | 0.64  (0.53-0.78) | 0.54  (0.44-0.66) | 0.91  (0.64-1.31) | 0.64  (0.44-0.95) | 0.64  (0.44-0.94) | 0.59  (0.40-0.88) | 1.01  (0.82-1.23) | 0.61  (0.48-0.76) | 0.64  (0.51-0.80) | 0.52  (0.41-0.65) |
|  | Oceania^a^ | 1.12  (0.86-1.46) | 1.12  (0.87-1.46) | 0.83  (0.63-1.09) | 0.96  (0.74-1.24) | 1.08  (0.65-1.80) | 1.05  (0.64-1.73) | 0.74  (0.43-1.27) | 0.98  (0.60-1.60) | 1.14  (0.84-1.55) | 1.15  (0.85-1.57) | 0.87  (0.63-1.20) | 0.94  (0.70-1.28) |
|  | ***(c) By country*** | | | | | | | | | | | | |
|  | Canada | 0.85  (0.73-1.00) | 0.99  (0.85-1.15) | 0.96  (0.83-1.12) | 0.43  (0.35-0.51) | 0.88  (0.63-1.24) | 1.01  (0.74-1.39) | 1.14  (0.85-1.53) | 0.45  (0.31-0.67) | 0.84  (0.70-1.01) | 0.98  (0.83-1.17) | 0.91  (0.76-1.08) | 0.42  (0.34-0.52) |
|  | USA | 0.92  (0.70-1.20) | 0.90  (0.69-1.17) | 0.81  (0.62-1.06) | 0.83  (0.64-1.07) | 0.77  (0.48-1.25) | 0.64  (0.39-1.05) | 0.64  (0.40-1.05) | 0.87  (0.56-1.35) | 1.00  (0.73-1.38) | 1.06  (0.77-1.44) | 0.92  (0.67-1.26) | 0.80  (0.58-1.11) |
|  | Denmark | 1.14  (0.71-1.83) | 0.47  (0.26-0.84) | 0.54  (0.31-0.94) | 0.36  (0.19-0.67) | 1.61  (0.55-4.68) | 0.79  (0.23-2.80) | 0.56  (0.14-2.18) | 0.84  (0.24-2.91) | 1.01  (0.60-1.71) | 0.38  (0.20-0.74) | 0.53  (0.29-0.98) | 0.24  (0.11-0.50) |
|  | France | 0.73  (0.49-1.08) | 0.24  (0.14-0.41) | 0.24  (0.14-0.41) | 0.16  (0.08-0.29) | 0.86  (0.39-1.93) | 0.28  (0.09-0.82) | 0.28  (0.10-0.81) | 0.19  (0.06-0.64) | 0.68  (0.44-1.07) | 0.23  (0.12-0.42) | 0.23  (0.12-0.42) | 0.14  (0.07-0.29) |
|  | The Netherlands | 0.98  (0.67-1.45) | 0.64  (0.41-0.98) | 0.45  (0.28-0.72) | 0.44  (0.28-0.71) | 0.78  (0.39-1.57) | 0.52  (0.24-1.13) | 0.36  (0.15-0.85) | 0.27  (0.10-0.69) | 1.11  (0.69-1.76) | 0.71  (0.42-1.19) | 0.50  (0.29-0.89) | 0.55  (0.32-0.95) |
|  | UK | 1.12  (0.86-1.47) | 0.90  (0.68-1.20) | 1.03  (0.79-1.34) | 0.91  (0.69-1.19) | 0.93  (0.53-1.64) | 0.90  (0.51-1.57) | 1.06  (0.63-1.78) | 0.99  (0.58-1.69) | 1.21  (0.89-1.64) | 0.90  (0.65-1.25) | 1.02  (0.75-1.38) | 0.86  (0.63-1.19) |
|  | Australia | 1.12  (0.86-1.46) | 1.12  (0.87-1.46) | 0.83  (0.63-1.09) | 0.96  (0.74-1.24) | 1.08  (0.65-1.80) | 1.05  (0.64-1.73) | 0.74  (0.43-1.27) | 0.98  (0.60-1.60) | 1.14  (0.84-1.55) | 1.15  (0.85-1.57) | 0.87  (0.63-1.20) | 0.94  (0.70-1.28) |
| **Female** | ***(a) Overall (7 countries including Canada, USA, 4 European countries and Australia)*** | | | | | | | | | | | | |
|  | Overall | 0.91  (0.81-1.02) | 0.94  (0.84-1.05) | 0.79  (0.71-0.89) | 0.63  (0.56-0.71) | 0.84  (0.66-1.06) | 0.84  (0.66-1.06) | 0.79  (0.63-1.00) | 0.61  (0.47-0.78) | 0.94  (0.83-1.07) | 0.99  (0.87-1.12) | 0.79  (0.70-0.90) | 0.64  (0.56-0.73) |
|  | ***(b) By continent*** | | | | | | | | | | | | |
|  | North America | 0.79  (0.67-0.92) | 0.86  (0.74-1.01) | 0.82  (0.70-0.95) | 0.52  (0.44-0.62) | 0.61  (0.44-0.84) | 0.71  (0.52-0.96) | 0.77  (0.58-1.02) | 0.50  (0.36-0.70) | 0.89  (0.74-1.06) | 0.95  (0.80-1.14) | 0.85  (0.71-1.01) | 0.53  (0.43-0.64) |
|  | Europe | 1.08  (0.89-1.32) | 0.91  (0.74-1.12) | 0.71  (0.57-0.88) | 0.71  (0.57-0.88) | 1.26  (0.82-1.95) | 0.87  (0.54-1.39) | 0.67  (0.40-1.10) | 0.71  (0.43-1.16) | 1.01  (0.82-1.25) | 0.93  (0.75-1.16) | 0.72  (0.57-0.91) | 0.72  (0.57-0.90) |
|  | Oceania^a^ | 1.09  (0.80-1.48) | 1.33  (1.00-1.77) | 0.88  (0.65-1.20) | 0.86  (0.63-1.17) | 1.30  (0.66-2.56) | 1.51  (0.81-2.81) | 1.15  (0.60-2.21) | 0.86  (0.42-1.75) | 1.02  (0.73-1.42) | 1.26  (0.92-1.73) | 0.79  (0.56-1.11) | 0.86  (0.62-1.20) |
|  | ***(c) By country*** | | | | | | | | | | | | |
|  | Canada | 0.80  (0.66-0.97) | 0.90  (0.75-1.09) | 0.90  (0.75-1.08) | 0.49  (0.39-0.61) | 0.52  (0.34-0.78) | 0.72  (0.50-1.04) | 0.82  (0.58-1.16) | 0.48  (0.32-0.72) | 0.97  (0.78-1.20) | 1.01  (0.82-1.24) | 0.94  (0.76-1.16) | 0.50  (0.39-0.63) |
|  | USA | 0.77  (0.58-1.03) | 0.79  (0.60-1.05) | 0.64  (0.48-0.85) | 0.57  (0.42-0.76) | 0.82  (0.49-1.38) | 0.72  (0.42-1.22) | 0.69  (0.41-1.15) | 0.57  (0.33-0.99) | 0.75  (0.54-1.04) | 0.84  (0.61-1.15) | 0.62  (0.44-0.86) | 0.57  (0.40-0.79) |
|  | Denmark | 0.97  (0.55-1.71) | 0.77  (0.43-1.37) | 0.53  (0.28-1.00) | 0.93  (0.54-1.60) | 1.09  (0.37-3.24) | 0.30  (0.06-1.44) | 0.43  (0.11-1.73) | 0.74  (0.23-2.36) | 0.91  (0.49-1.71) | 1.02  (0.56-1.87) | 0.58  (0.30-1.15) | 1.02  (0.56-1.87) |
|  | France | 0.76  (0.48-1.20) | 0.32  (0.18-0.56) | 0.23  (0.12-0.44) | 0.27  (0.14-0.50) | 1.03  (0.44-2.45) | 0.24  (0.07-0.87) | 0.29  (0.09-0.96) | 0.39  (0.12-1.22) | 0.63  (0.38-1.05) | 0.35  (0.19-0.65) | 0.21  (0.10-0.42) | 0.21  (0.10-0.43) |
|  | The Netherlands | 0.79  (0.54-1.16) | 0.70  (0.47-1.04) | 0.38  (0.24-0.61) | 0.39  (0.24-0.61) | 0.73  (0.28-1.87) | 1.02  (0.44-2.36) | 0.37  (0.12-1.17) | 0.41  (0.14-1.23) | 0.81  (0.54-1.22) | 0.60  (0.38-0.94) | 0.38  (0.23-0.64) | 0.38  (0.23-0.63) |
|  | UK | 1.74  (1.27-2.40) | 1.70  (1.24-2.34) | 1.50  (1.10-2.05) | 1.36  (0.98-1.87) | 2.46  (1.17-5.20) | 1.83  (0.84-3.98) | 1.63  (0.75-3.55) | 1.51  (0.68-3.37) | 1.52  (1.09-2.12) | 1.67  (1.19-2.33) | 1.46  (1.06-2.03) | 1.31  (0.94-1.83) |
|  | Australia | 1.09  (0.80-1.48) | 1.33  (1.00-1.77) | 0.88  (0.65-1.20) | 0.86  (0.63-1.17) | 1.30  (0.66-2.56) | 1.51  (0.81-2.81) | 1.15  (0.60-2.21) | 0.86  (0.42-1.75) | 1.02  (0.73-1.42) | 1.26  (0.92-1.73) | 0.79  (0.56-1.11) | 0.86  (0.62-1.20) |

^a^ Oceania includes Australia only.
