# Supplementary material for: Correction: Anal cancer in high-income countries: Increasing burden of disease
Source: PLoS One. 2019 May 8;14(5):e0216884. doi: 10.1371/journal.pone.0216884 (PMC6505931; doi:10.1371/journal.pone.0216884)
Supplement: S2 Fig — (DOCX) [file pone.0216884.s001.docx]

S2 Fig. Pooled age-specific incidence of adenocarcinoma of the anus by birth cohort in men and women born from 1900 to 1988, for each of the five 5-yearly average rates (1988-92, 1993-97, 1998-2002, 2003-2007, 2008-2012)
